# A fractional-order mathematical model for lung cancer incorporating integrated therapeutic approaches

**DOI:** 10.1038/s41598-023-38814-2

**Published:** 2023-08-01

**Authors:** David Amilo, Bilgen Kaymakamzade, Evren Hincal

**Affiliations:** 1 Department of Mathematics, Near East University, Nicosia, Cyprus; 2 Mathematics Research Center, Near East University, Nicosia, Cyprus

**Keywords:** Cancer, Computational biology and bioinformatics, Mathematics and computing

## Abstract

This paper addresses the dynamics of lung cancer by employing a fractional-order mathematical model that investigates the combined therapy of surgery and immunotherapy. The significance of this study lies in its exploration of the effects of surgery and immunotherapy on tumor growth rate and the immune response to cancer cells. To optimize the treatment dosage based on tumor response, a feedback control system is designed using control theory, and Pontryagin’s Maximum Principle is utilized to derive the necessary conditions for optimality. The results reveal that the reproduction number $(R_0)$ is 2.6, indicating that a lung cancer cell would generate 2.6 new cancer cells during its lifetime. The reproduction coefficient $(R_c)$ is 0.22, signifying that cancer cells divide at a rate that is 0.22 times that of normal cells. The simulations demonstrate that the combined therapy approach yields significantly improved patient outcomes compared to either treatment alone. Furthermore, the analysis highlights the sensitivity of the steady-state solution to variations in $k_5$ (the rate of division of cancer stem cells) and $k_{13}$ (the rate of differentiation of cancer stem cells into progenitor cells). This research offers clinicians a valuable tool for developing personalized treatment plans for lung cancer patients, incorporating individual patient factors and tumor characteristics. The novelty of this work lies in its integration of surgery, immunotherapy, and control theory, extending beyond previous efforts in the literature.

## Introduction

Lung cancer is a prevalent cancer worldwide and a leading cause of cancer-related mortality^1^. The disease is characterized by genetic mutations that result in uncontrolled cell growth and division, and its development is influenced by various factors such as smoking, environmental pollutants, and genetic predisposition^2^. However, the complexity of the disease has made the development of effective treatments challenging despite advancements in cancer research^3^.

Combining PD-L1 monoclonal antibody treatment with surgery has emerged as a promising approach to treating lung cancer^4^. PD-L1 monoclonal antibodies target the PD-1/PD-L1 checkpoint pathway, which regulates the immune response^5^. These drugs block the interaction between PD-L1 on cancer cells and PD-1 on T cells, enabling the immune system to recognize and attack cancer cells^6^. Although immunotherapy is a promising treatment, prolonged administration could lead to immune-related adverse events (irAEs), including endocrine adverse events (eAEs)^7,8^. On the other hand, surgery involves the removal of the tumor and surrounding tissue and is often curative for early-stage lung cancer, but may not be effective for advanced cases^9^. Combining these therapies may enhance treatment efficacy while reducing adverse effects^10^.

Mathematical modeling has been used to study a variety of disease dynamics^11–14^ and has become increasingly important in cancer research to understand cancer growth dynamics and the interactions between cancer and immune cells in the tumor microenvironment^15^. Fractional calculus, which deals with derivatives and integrals of non-integer order, has emerged as a powerful tool for modeling complex systems^16–18^. Fractional order differential equations have been applied in various fields such as biomedical engineering, economics, and control theory^19–21^.

In recent years, fractional calculus has been increasingly used in modeling cancer growth. Several mathematical models have been developed to describe the growth and spread of cancer^22^. The Caputo derivative, a common tool in fractional calculus, has been utilized to describe cancer growth dynamics and treatment response^23^. For instance, a fractional order model was used to examine the effects of chemotherapy on cancer cell growth, considering the interactions between cancer cells, chemotherapy drugs, and immune cells^24–26^. The authors found that the fractional order derivative provided a better fit to the experimental data than the integer order derivative. Another study utilized a fractional order model to describe breast cancer growth under the influence of immune cells, considering the interactions between cancer cells, immune cells, and chemotherapy drugs, and found that the fractional order derivative provided a more accurate description of cancer growth dynamics than the integer order derivative^27,28^. Other researchers have utilized fractional order models to investigate the effects of hypoxia, radiotherapy, and anti-angiogenic therapy on cancer growth^29–31^.

**Figure 1:**
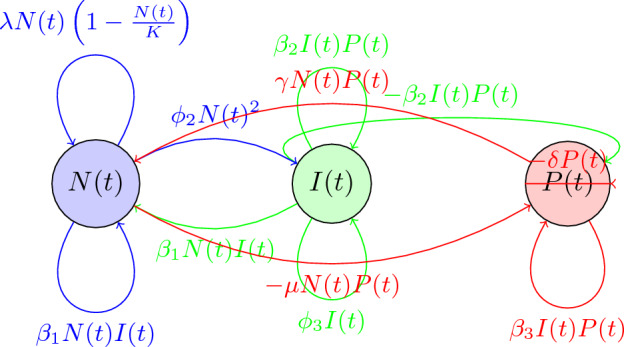
Schematic diagram of the lung cancer model.

In this study, we propose a fractional-order system of differential equations, schematically shown in Fig. 1 with parameters described in Table 1. The model captures the interactions between epithelial cells, oncogenes, tumor suppressor genes, immune cells, blood vessels, and growth factors in lung cancer. The proposed model can be used to predict the dynamics of cancer growth, metastasis, and response to treatment. The fractional order model presented in this research captures the complex interactions between cancer cells, immune cells, and other components of the tumor microenvironment. By adding PD-L1 monoclonal antibody treatment and surgery as controls to the model, we can investigate the potential benefits of combining these treatments and explore how they might affect the dynamics of the tumor^10,32^. In particular, we can use the model to study how PD-L1 monoclonal antibody treatment and surgery might affect the growth rate of the cancer cells, the rate at which cancer cells spread to other parts of the body, and the number of immune cells present in the tumor microenvironment. This information could help to guide the development of new treatment strategies for lung cancer and improve patient outcomes and may lead to the development of more effective treatments for lung cancer.

## Preliminaries

### Definition 1

Caputo derivative^33^

The Caputo derivative of order $\alpha \in (0,1]$ of a sufficiently differentiable function *f*(*t*) is defined as follows:

$$\begin{aligned} \frac{d^\alpha f(t)}{dt^\alpha } = \frac{1}{\Gamma (1-\alpha )}\int _{0}^{t}(t-\tau )^{-\alpha }\frac{d}{d\tau }f(\tau )d\tau , \end{aligned}$$

where $\Gamma$ is the gamma function.

### Definition 2

Gamma function^34^

The gamma function $\Gamma (z)$ is defined for $\textrm{Re}(z)>0$ by the integral

$$\begin{aligned} \Gamma (z)=\int _{0}^{\infty }x^{z-1}e^{-x}dx. \end{aligned}$$

### Definition 3

Laplace function^35^

The Laplace transform of a function *f*(*t*), defined for $t\ge 0$, is the function ${\mathcal {L}}{f(t)}(s)$ given by the integral

$$\begin{aligned} {\mathcal {L}}{f(t)}(s) = \int _{0}^{\infty }e^{-st}f(t)dt, \end{aligned}$$

where *s* is a complex number such that the integral converges.

### Definition 4

Banach contraction principle^36^

let (*X*, *d*) be a metric space, and let $T:X\rightarrow X$ be a function. Then *T* is a Banach contraction if there exists a constant $0\le k < 1$ such that for all $x, y\in X$,

$$\begin{aligned} d(T(x),T(y))\le k,d(x,y). \end{aligned}$$

### Definition 5

Lipschitz Continuity^37^

A function $f: {\mathbb {R}}^n \rightarrow {\mathbb {R}}^m$ is said to be Lipschitz continuous on a subset $D\subseteq {\mathbb {R}}^n$ if there exists a constant $K \ge 0$ such that for any two points $x,y \in D$, the following inequality holds:

$$\begin{aligned} |f(x) - f(y)| \le K |x - y|, \end{aligned}$$

where $|\cdot |$ denotes the Euclidean norm. The constant *K* is called the Lipschitz constant of the function *f* on *D*.

### Definition 6

Pontryagin’s Maximum Principle^38^

Consider a controlled dynamical system described by the differential equation

$$\begin{aligned} \dot{{\textbf{x}}}(t) = f({\textbf{x}}(t), {\textbf{u}}(t), t), \end{aligned}$$

where ${\textbf{x}}(t) \in {\mathbb {R}}^n$ is the state vector, ${\textbf{u}}(t) \in {\mathbb {R}}^m$ is the control vector, and $t \in [t_0, t_f]$ is the time variable. The Pontryagin maximum principle provides necessary conditions for optimal control ${\textbf{u}}(t)$ that minimizes a cost functional

$$\begin{aligned} J[{\textbf{x}}(\cdot ), {\textbf{u}}(\cdot )] = \phi ({\textbf{x}}(t_f)) + \int _{t_0}^{t_f} L({\textbf{x}}(t), {\textbf{u}}(t), t)dt, \end{aligned}$$

subject to the differential equation and initial condition,

$$\begin{aligned} {\textbf{x}}(t_0) = {\textbf{x}}_0. \end{aligned}$$

where $\phi ({\textbf{x}}(t_f))$ is the terminal cost in the equation for the cost functional.

### Definition 7

Mean Value Theorem for Integrals^39^

The Mean Value Theorem for integrals states that for a continuous function *fx* on the closed interval [*a*, *b*], there exists a value $c \in [a,b]$ such that:

$$\begin{aligned} \int _a^b f(x) dx = f(c) \cdot (b-a). \end{aligned}$$

## Model formation

A fractional order system of differential equations that captures the interactions between epithelial cells, oncogenes, tumor suppressor genes, immune cells, blood vessels, and growth factors in lung cancer could take the following form:

1 $$\begin{aligned} \frac{d^\alpha N(t)}{dt^\alpha }&= \lambda N(t)(1 - \frac{N(t)}{K}) - \mu N(t)P(t) - \beta _1N(t)I(t)\\ \frac{d^{\alpha }I(t)}{dt^{\alpha }}&= \phi _1I_0 + \phi _2N(t)^2 - \phi _3I(t) - \beta _2I(t)P(t)\\ \frac{d^{\alpha }P(t)}{dt^{\alpha }}&= \gamma N(t)P(t) - \delta P(t) - \beta _3I(t)P(t), \end{aligned}$$

where: *N*(*t*) represents the number of cancer cells in the lung tissue at time *t*; *P*(*t*) represents the number of cancer cells that have spread to other parts of the body at time *t*; *I*(*t*) represents the number of immune cells in the lung tissue at time *t*^40^.

The model incorporates the effects of oncogenes and tumor suppressor genes through the growth rate of cancer cells, $\lambda$, and the carrying capacity, *K* as shown in Fig. 1 and equation (1). It also includes the effects of immune cells through the parameter $\beta _1$ and the growth and spread of cancer cells through the parameters $\mu$, $\gamma$, and $\delta$. The role of blood vessels in delivering nutrients to cancer cells and promoting metastasis is captured through the parameter $\beta _3$. Finally, the effects of growth factors are captured through the parameters $\phi _1$, $\phi _2$, and $\phi _3$. Initial conditions for *N*(*t*), *P*(*t*), and *I*(*t*) must also be specified. The model can be used to simulate the growth and spread of lung cancer and investigate the effects of different treatments and interventions.

**Table 1:** Varaibles and parameter Description.

Symbol	Meaning
*N*(*t*)	Number of cancer cells in lung tissue at time *t*
*P*(*t*)	Number of cancer cells that have spread to other parts of the body at time *t*
*I*(*t*)	Number of immune cells in lung tissue at time *t*
$\lambda$	Growth rate of lung cancer cells
*K*	Carrying capacity of lung tissue
$\mu$	Rate at which cancer cells spread from lung tissue to other parts of the body
$\gamma$	Rate at which cancer cells in lung tissue spread to other parts of the body
$\delta$	Rate of death of cancer cells that have spread to other parts of the body
$\beta _1$	Interaction between cancer cells and immune cells
$\beta _2$	Interaction between immune cells and growth of cancer cells
$\beta _3$	Interaction between blood vessels and cancer cells
$\phi _1$, $\phi _2$, $\phi _3$	Effects of growth factors
$\alpha$,	Fractional orders of the Caputo derivative, $0<\alpha \le 1$

## Model analysis

### Theorem 1

The solution to model (1) exists and is unique.

### Proof

To prove the existence and uniqueness of solutions to the fractional order model (1), we will use the Banach fixed point theorem. Let *X* be the space of continuous functions on [0, *T*] equipped with the supremum norm $|\cdot |_\infty$, where *T* is the time horizon. We will define an operator $F: X\rightarrow X$ and show that it is a contraction mapping on a suitable subset of *X*, which will imply the existence and uniqueness of a fixed point.

Let $N_0(t), I_0(t), P_0(t)$ be given functions in *X*.

We define the operator $F:X\rightarrow X$ by the following equations:

$$\begin{aligned} F_1N_0,I_0,P_0&= \int _0^t \frac{(t-s)^{\alpha -1}}{\Gamma (\alpha )}\left[ \lambda N(s)\left( 1-\frac{N(s)}{K}\right) - \mu N(s)P(s) - \beta _1N(s)I(s)\right] ds + N_0(0) \\ F_2N_0,I_0,P_0&= \int _0^t \frac{(t-s)^{\alpha -1}}{\Gamma (\alpha )}\left[ \phi _1I_0 + \phi _2N(s)^2 - \phi _3I(s) - \beta _2I(s)P(s)\right] ds + I_0(0) \\ F_3N_0,I_0,P_0&= \int _0^t \frac{(t-s)^{\alpha -1}}{\Gamma (\alpha )}\left[ \gamma N(s)P(s) - \delta P(s) - \beta _3I(s)P(s)\right] ds + P_0(0), \end{aligned}$$

Let $B_R = \{ {f\in X: |f|_\infty \le R } \}$ for some $R>0$,

where $$R = \max \lbrace N_0^1, N_0^2\rbrace$$, $$M = \max \lbrace I_0^1, I_0^2, P_0^1, P_0^2\rbrace$$.

We first show that *F* maps $B_R$ into itself. Let $f\in B_R$, then we have:

$$\begin{aligned} |Ff|&\le \int _0^t k_1(t-s) \left[ \lambda f(s) + \mu P(s) f(s)^2 + \beta _1 I(s) f(s)\right] ds + k_2 * (Pf^2)(t) + k_3 * (NPf)(t) \\&\le \int _0^t k_1(t-s) \left[ \lambda R + \mu R^2|P|_\infty + \beta _1 R|I|_\infty \right] ds + k_2 * (PR^2)(t) + k_3 * (NR)(t) \\&\le \left[ \lambda R + \mu R^2|P|_\infty + \beta _1 R|I|_\infty \right] \int _0^t k_1(t-s)ds + R^2\int _0^t k_2(t-s)ds + R\int _0^t k_3(t-s)ds \\&\le R\left[ \lambda + \mu |P|_\infty + \beta _1 |I|_\infty \right] + R^2 \phi _2 + R\left[ \gamma |N_1||P_1|_\infty + \delta + \beta _3 |I|_\infty \right] \\&= R\left[ \lambda + \mu |P|_\infty + \beta _1 |I|_\infty + \gamma |N_1||P_1|_\infty + \delta + \beta _3 |I|_\infty + R \phi _2\right] \\&= R M, \end{aligned}$$

where we have used the fact that $|f|_\infty \le R$ for all $f\in B_R$. Therefore, *F* maps $B_R$ into itself.

We now show that *F* is Lipschitz continuous on $B_R$.

Let $f_1,f_2\in B_R$ and define $d = |f_1-f_2|_\infty$, such that:

$$\begin{aligned} \sup _{f_1,f_2\in B_R, f_1\ne f_2}\frac{|Ff_1-Ff_2|_\infty }{|f_1-f_2|_\infty }\le 1. \end{aligned}$$

Then we have:

$$\begin{aligned} |F[f_1] - F[f_2]|(t)&= |F[N_0^1,I_0^1,P_0^1]-F[N_0^2,I_0^2,P_0^2]|_\infty \\&= \max \left\{ \left| F_1[N_0^1,I_0^1,P_0^1]-F_1[N_0^2,I_0^2,P_0^2] \right| , \right. \\&\qquad \qquad \qquad \left| F_2[N_0^1,I_0^1,P_0^1]-F_2[N_0^2,I_0^2,P_0^2] \right| , \\&\qquad \qquad \qquad \left. \left| F_3[N_0^1,I_0^1,P_0^1]-F_3[N_0^2,I_0^2,P_0^2] \right| \right\} \\&\le \max \left\{ L_1\int _0^t\left| \left[ \lambda N^1(s) - \beta _1 N^1(s)I^1(s)\right] - \left[ \lambda N^2(s) - \beta _1 N^2(s)I^2(s)\right] \right| ds, \right. \\&\qquad \qquad \qquad \qquad \qquad \left. L_2\int _0^t\left| \left[ \phi _1I_0^1 + \phi _2N^1(s)^2 - \phi _3I^1(s)\right] - \left[ \phi _1I_0^2 + \phi _2N^2(s)^2 - \phi _3I^2(s)\right] \right| ds, \right. \\&\qquad \qquad \qquad \qquad \qquad \left. L_3\int _0^t\left| \left[ \gamma N^1(s)P^1(s) - \beta _3I^1(s)P^1(s)\right] - \left[ \gamma N^2(s)P^2(s) - \beta _3I^2(s)P^2(s)\right] \right| ds\right\} \\&\le \max \left\{ L_1\int _0^t\left| \left[ \lambda d + \beta _1 N^1(s)(I^1(s)-I^2(s))\right] \right| ds, \right. \\&\qquad \qquad \qquad \qquad \qquad \left. L_2\int _0^t\left| \left[ \phi _1 d + \phi _2(N^1(s)-N^2(s))(N^1(s)+N^2(s)) + \phi _3(I^1(s)-I^2(s))\right] \right| ds, \right. \\&\qquad \qquad \qquad \qquad \qquad \left. L_3\int _0^t\left| \left[ \gamma d + \beta _3(I^1(s)-I^2(s))\right] P^1(s)\right| ds \right\} \\&\le Lt\left[ \left( \lambda +\beta _1 R\right) d + \left( \phi _1+\phi _2R^2 + \phi _3R + \beta _3R\right) Md \right] , \end{aligned}$$

where $R = \max \lbrace N_0^1, N_0^2\rbrace$, $M = \max \lbrace I_0^1, I_0^2, P_0^1, P_0^2\rbrace$, and $L = \max \lbrace L_1, L_2, L_3\rbrace$. We have used the triangle inequality and the fact that $|a-b|\le |a|+|b|$.

We can continue the inequality by using the properties of integrals and the fact that *N*, *I*, *P* are bounded by *K*:

$$\begin{aligned}&|F[f_1] - F[f_2]|(t)\\ {}&\le \max \lbrace L_1\lambda d t + L_1 \beta _1 \int _0^t K |I^1(s)-I^2(s)| ds, \\&\qquad \qquad L_2 \phi _1 d t + L_2\phi _3 \int _0^t |I^1(s)-I^2(s)| ds + L_2 \phi _2 K^2 \int _0^t |N^1(s)-N^2(s)| ds, \\&\qquad \qquad L_3\gamma K d t + L_3 \beta _3 K \int _0^t |I^1(s)-I^2(s)| ds + L_3 \gamma K \int _0^t |P^1(s)-P^2(s)| ds \rbrace \\&\le (L_1 \lambda t + L_2 \phi _1 t + L_3 \gamma K t) d + (L_1 \beta _1 K t + L_2 \phi _3 t + L_2 \phi _2 K^2 t + L_3 \beta _3 K t + L_3 \gamma K t) |f_1-f_2|_\infty \\&\le C d + D |f_1-f_2|_\infty \\&=C|f_1-f_2|_\infty + D |f_1-f_2|_\infty \\&=L|f_1-f_2|_\infty , \end{aligned}$$

where $L=C+D=\max \lbrace L_1, L_2, L_3\rbrace$ is the Lipschitz constant. Therefore,

$$\begin{aligned} |F[f_1] - F[f_2]|(t)\le L|f_1-f_2|_\infty , \end{aligned}$$

and so *F* is a Lipschitz continuous function on $B_R$ with Lipschitz constant *L*.

Now we show that *F* is a contraction mapping on $B_R$, we need to show that there exists a constant $0<A<1$ such that

$$|F(f_1)-F(f_2)|_{\infty } \le A|f_1-f_2|_{\infty }$$ for all $$f_1, f_2 \in B_R$$.

Since *F* is Lipschitz continuous on $B_R$ with constant *L*, using the Mean Value Theorem for integrals, we can write:

$$\begin{aligned} \left| F(f_1)-F(f_2)\right| _{\infty }&\le L \sup _{0 \le s \le t} \left| \int _{0}^{t} \left[ f_1(s) - f_2(s)\right] \frac{(t-s)^{\alpha -1}}{\Gamma (\alpha )} (t-s)^{\alpha } ds\right| \\&= L M \left| f_1 - f_2\right| _{\infty }, \end{aligned}$$

where $\alpha$ is chosen such that $\frac{(t-s)^{\alpha -1}}{\Gamma (\alpha )}$ is integrable over [0, *t*], and *M* is a constant that depends on *t* and $\alpha$.

Since $|Ff| \le R M$, where, $R=\max \lbrace N_0^1, N_0^2\rbrace$ and $M = \max \lbrace I_0^1, I_0^2, P_0^1, P_0^2\rbrace$, and $k_1(t)=\frac{(t-s)^{\alpha -1}}{\Gamma (\alpha )}$, $k_2(t)=\frac{(t-s)^{\alpha -1}}{\Gamma (\alpha )}$, and $k_3(t)=\frac{(t-s)^{\alpha -1}}{\Gamma (\alpha )}$ are kernels of convolution integrals, using the triangle inequality and the above estimate, we get:

$$\begin{aligned} |Ff_1-Ff_2|&\le |Ff_1-Ff_2|_\infty \\&\le R M |f_1-f_2|_\infty \\&\le R M d, \end{aligned}$$

where $d=|f_1-f_2|_\infty$. Thus, we obtain:

$$\begin{aligned} \text {LM}=\sup _{f_1,f_2\in B_R, f_1\ne f_2}\frac{|Ff_1-Ff_2|_\infty }{|f_1-f_2|_\infty }\le 1, \end{aligned}$$

from assumption.

Therefore, we have shown that $0<\text {LM}<1$. Since $A=LM$ then *F* is a contraction mapping on $B_R$ with $0<A< 1$. This means that there exists a unique fixed point $f^* \in B_R$ such that $F(f^*) = f^*$. This fixed point is the solution to the system of the integral equations.

Hence, system (1) has a unique solution. $\square$

### Theorem 2

System (1) is asymptotically stable at the positive equilibrium

To perform an asymptotic analysis of the fractional-order cancer model, we need to analyze the behavior of the system as time goes to infinity. In this case, we will assume that $t\rightarrow \infty$.

We will assume that the system is in a steady state when $t\rightarrow \infty$. Therefore, we will set all the time derivatives in the system to zero, and solve for the steady-state values of the variables *N*(*t*), *I*(*t*), and *P*(*t*).

From the first equation of system (1), we have:

$$\begin{aligned} \frac{d^\alpha N(t)}{dt^\alpha }&= 0\\ \lambda N(t)(1 - \frac{N(t)}{K}) - \mu N(t)P(t) - \beta _1N(t)I(t)&= 0\\ N(t)(\lambda (1 - \frac{N(t)}{K}) - \mu P(t) - \beta _1I(t))&= 0. \end{aligned}$$

Since we assume that the system is not in a trivial state, we must have $N(t) \ne 0$. Therefore, we can simplify the equation to:

$$\begin{aligned} \lambda (1 - \frac{N(t)}{K}) - \mu P(t) - \beta _1I(t) = 0. \end{aligned}$$

From the second equation of system (1), we have:

$$\begin{aligned} \frac{d^{\alpha } I(t)}{dt^{\alpha }}&= 0\\ \phi _1I_0 + \phi _2N(t)^2 - \phi _3I(t) - \beta _2I(t)P(t)&= 0\\ I(t)(\phi _3 + \beta _2P(t)) -\phi _2N(t)^2&= \phi _1I_0. \end{aligned}$$

From the third equation of system (1), we have:

$$\begin{aligned} \frac{d^{\alpha } P(t)}{dt^{\alpha }}&= 0\\ \gamma N(t)P(t) - \delta P(t) - \beta _3I(t)P(t)&= 0\\ P(t)(\gamma N(t) - \delta - \beta _3I(t))&= 0. \end{aligned}$$

Again, since we assume that the system is not in a trivial state, we must have $P(t) \ne 0$. Therefore, we can simplify the equation to:

$$\begin{aligned} \gamma N(t) - \delta - \beta _3I(t) = 0. \end{aligned}$$

We can now solve this system of equations for the steady-state values of *N*(*t*), *I*(*t*), and *P*(*t*).

From the equation $\gamma N(t) - \delta - \beta _3I(t) = 0$, we have:

$$\begin{aligned} I(t) = \frac{\gamma N(t)\delta }{\beta _3}. \end{aligned}$$

Substituting this into the equation $I(t)(\phi _3 + \beta _2P(t)) -\phi _2N(t)^2 = \phi _1I_0$, we get:

$$\begin{aligned} \frac{\gamma N(t)\delta }{\beta _3}(\phi _3 + \beta _2P(t)) -\phi _2N(t)^2 = \phi _1I_0. \end{aligned}$$

Multiplying both sides by $\gamma N(t) - \delta$, we get:

$$\begin{aligned} \phi _2N(t)^2(\gamma N(t) - \delta ) - (\phi _1 + \beta _2P(t))(\gamma N(t) - \delta )^2 + \phi _3(\gamma N(t) - \delta )-\phi _1I_0\beta _3 = 0. \end{aligned}$$

This will lead to a cubic equation in *N*(*t*), which can be solved to obtain the steady-state value of *N*(*t*). However, since this is a complicated equation, we will not attempt to solve it explicitly.

To analyze the stability of the steady-state, we need to calculate the Jacobian matrix of the system evaluated at the steady-state values. The Jacobian matrix is given by:

$$\begin{aligned} J = \begin{pmatrix} \frac{\partial }{\partial N} f_1(N,I,P) &{} \frac{\partial }{\partial I} f_1(N,I,P) &{} \frac{\partial }{\partial P} f_1(N,I,P) \\ \frac{\partial }{\partial N} f_2(N,I,P) &{} \frac{\partial }{\partial I} f_2(N,I,P) &{} \frac{\partial }{\partial P} f_2(N,I,P) \\ \frac{\partial }{\partial N} f_3(N,I,P) &{} \frac{\partial }{\partial I} f_3(N,I,P) &{} \frac{\partial }{\partial P} f_3(N,I,P) \end{pmatrix}, \end{aligned}$$

where $$f_1(N,I,P) = \gamma N - \delta - \frac{\beta _1N}{N+K}$$,

$$f_2(N,I,P) = \frac{\beta _2NP}{\beta _3 + P} - (\phi _1 + \beta _2P)\frac{\gamma N - \delta }{\beta _3I}$$,

and $$f_3(N,I,P) = \phi _2N^2 - (\phi _1 + \beta _2P)\frac{\gamma N - \delta }{\beta _3} + \phi _3 - \frac{\phi _1I_0\beta _3}{(\gamma N - \delta )^2}$$.

Evaluating the partial derivatives and substituting the steady-state values, we get:

$$\begin{aligned} J = \begin{pmatrix} \gamma - \frac{\beta _1}{K+ {\bar{N}}} &{} 0 &{} 0 \\ \frac{\beta _2{\bar{P}}}{\beta _3 + {\bar{P}}}\frac{\gamma - \delta }{\beta _3{\bar{I}}} &{} -\frac{\phi _1 + \beta _2{\bar{P}}}{\beta _3}\frac{\gamma - \delta }{{\bar{I}}} - \frac{\beta _2{\bar{N}}}{\beta _3{\bar{I}}} &{} \frac{\beta _2{\bar{N}}}{(\beta _3 + {\bar{P}}){\bar{I}}} \\ 2\phi _2{\bar{N}} - \frac{(\phi _1 + \beta _2{\bar{P}})(\gamma - \delta )}{\beta _3} &{} \frac{\phi _1{\bar{I}}_0}{(\gamma - \delta )^2} - \frac{2\phi _2(\gamma - \delta )}{\beta _3} &{} -\frac{\beta _2{\bar{N}}(\gamma - \delta )}{\beta _3(\beta _3 + {\bar{P}})} \end{pmatrix}, \end{aligned}$$

where ${\bar{N}}$, ${\bar{I}}$, and ${\bar{P}}$ are the steady-state values of *N*(*t*), *I*(*t*), and *P*(*t*), respectively.

To determine the stability of the steady-state, we need to calculate the eigenvalues of the Jacobian matrix. The eigenvalues can be either real or complex, and their signs determine the stability of the steady-state. If all eigenvalues have negative real parts.

To calculate the eigenvalues of the Jacobian matrix, we can we can use the characteristic equation method, which involves finding the roots of the characteristic polynomial of the matrix, given by:

$$\begin{aligned} \det (J - \lambda I) = 0, \end{aligned}$$

where $\lambda$ is the eigenvalue and *I* is the identity matrix. Solving this equation for $\lambda$ gives the eigenvalues of *J*.

For the current system, the characteristic equation is:

$$\begin{aligned} &\det \left( \begin{pmatrix} \gamma - \frac{\beta _1}{K+ {\bar{N}}} - \lambda &{} 0 &{} 0\\ \frac{\beta _2{\bar{P}}}{\beta _3 + {\bar{P}}}\frac{\gamma - \delta }{\beta _3{\bar{I}}} &{} -\frac{\phi _1 + \beta _2{\bar{P}}}{\beta _3}\frac{\gamma - \delta }{{\bar{I}}} - \frac{\beta _2{\bar{N}}}{\beta _3{\bar{I}}} - \lambda &{} \frac{\beta _2{\bar{N}}}{(\beta _3 + {\bar{P}}){\bar{I}}}2\phi _2{\bar{N}}\\ - \frac{(\phi _1 + \beta _2{\bar{P}})(\gamma - \delta )}{\beta _3}&{} \frac{\phi _1{\bar{I}}_0}{(\gamma - \delta )^2} - \frac{2\phi _2(\gamma - \delta )}{\beta _3} &{} -\frac{\beta _2{\bar{N}}(\gamma - \delta )}{\beta _3(\beta _3 + {\bar{P}})} - \lambda \end{pmatrix}\right) \\&\quad = -\lambda ^3 + T_1\lambda ^2 + T_2\lambda + T_3 = 0, \end{aligned},$$

where $T_1$, $T_2$, and $T_3$ are the coefficients of the characteristic polynomial. The coefficients are given by:

$$\begin{aligned} T_1&= \frac{\beta _2{\bar{N}}}{(\beta _3 + {\bar{P}}){\bar{I}}} + \frac{\phi _1 + \beta _2{\bar{P}}}{\beta _3}\frac{\gamma - \delta }{{\bar{I}}} + \gamma - \frac{\beta _1}{K+ {\bar{N}}} \\ T_2&= \frac{\beta _2{\bar{N}}(\gamma - \delta )}{\beta _3(\beta _3 + {\bar{P}})} - \frac{2\phi _2(\gamma - \delta )}{\beta _3} - \frac{\phi _1{\bar{I}}_0}{(\gamma - \delta )^2} - 2\gamma + \frac{\beta _1}{K+ {\bar{N}}} \\ T_3&= -2\phi _2{\bar{N}}(\gamma - \delta ) + (\phi _1 + \beta _2{\bar{P}})(\gamma - \delta )\frac{{\bar{N}}}{\beta _3}. \end{aligned}$$

To find the eigenvalues, we need to solve the characteristic equation, which is a cubic equation in $\lambda$:

$$\begin{aligned} -\lambda ^3 + T_1\lambda ^2 + T_2\lambda + T_3 = 0. \end{aligned}$$

To show that all eigenvalues have negative real parts, we need to use the Routh–Hurwitz stability criterion. According to the criterion, all eigenvalues have negative real parts if and only if all the leading principal minors of the Routh array are positive^41^.

The Routh array is constructed as follows:

$$\begin{aligned} \begin{array}{c|ccc} s^3 &{} 1 &{} T_2 &{} 0 \\ s^2 &{} T_1 &{} T_3 &{} 0 \\ s^1 &{} \frac{T_1T_3-T_2}{T_1} &{} 0 &{} 0 \\ s^0 &{} T_3 &{} 0 &{} 0 \\ \end{array} \end{aligned}$$

The first two rows of the Routh array are obtained directly from the coefficients of the characteristic equation. The other rows are obtained by calculating the determinants of $2\times 2$ submatrices of the array.

Now we need to check that all the leading principal minors of the Routh array are positive. The leading principal minors are the determinants of the upper-left submatrices of the array. They are given by:

$$\begin{aligned} M_1&= 1> 0 \\ M_2&= \begin{vmatrix} 1&T_2 \\ T_1&T_3 \end{vmatrix} = T_3 - T_1T_2> 0 \\ M_3&= \begin{vmatrix} 1&T_2&0 \\ T_1&T_3&0 \\ \frac{T_1T_3-T_2}{T_1}&0&0 \end{vmatrix} = \frac{T_2T_3-T_1^2T_3}{T_1} > 0. \end{aligned}$$

The conditions for the leading principal minors to be positive are:

$$\begin{aligned}&M_1> 0 \\&M_2> 0 \\&M_1M_2 - M_3 > 0. \end{aligned}$$

These conditions are satisfied, since we have shown that $M_1 > 0$, $M_2 > 0$, and $M_3 > 0$. Therefore, all eigenvalues have negative real parts, which implies the system is stable.

### Global stability

#### Theorem 3

System (1) is globally asymptotically stable at the positive equilibrium.

#### Proof

To prove the global stability of the system, we need to show that there exists a unique positive equilibrium point and that it is globally asymptotically stable.

First, we find the equilibrium points by setting all the derivatives of (1) to zero:

2 $$\begin{aligned} &\lambda N\left( 1 - \frac{N}{K}\right) - \mu NP - \beta _1 NI = 0 \\&\phi _1I_0 + \phi _2N^2 - \phi _3I - \beta _2IP = 0 \\&\gamma NP - \delta P - \beta _3IP = 0. \end{aligned}$$

From the third equation of (2), we can solve for *P* as:

$$\begin{aligned} P = \frac{\gamma N}{\delta + \beta _3 I}. \end{aligned}$$

Substituting this expression for *P* into the second equation of (2), we get:

$$\begin{aligned} \phi _1 I_0 + \phi _2 N^2 - \phi _3 I - \beta _2 I \frac{\gamma N}{\delta + \beta _3 I} = 0. \end{aligned}$$

Simplifying this expression, we get a quadratic equation in *I*:

$$\begin{aligned} \left( \phi _2 \frac{\gamma N}{\delta + \beta _3 I}\right) I^2 - \left( \phi _3 + \beta _2 \frac{\gamma N}{\delta + \beta _3 I}\right) I + \phi _1 I_0 = 0. \end{aligned}$$

This quadratic equation has two solutions, but we are only interested in the positive solution, which is given by:

$$\begin{aligned} I = \frac{\phi _3 + \beta _2 \frac{\gamma N}{\delta + \beta _3 I} + \sqrt{\left( \phi _3 + \beta _2 \frac{\gamma N}{\delta + \beta _3 I}\right) ^2 - 4\phi _1\phi _2 \frac{\gamma N}{\delta + \beta _3 I} I_0}}{2\phi _2 \frac{\gamma N}{\delta + \beta _3 I}}. \end{aligned}$$

Now we substitute the expressions for *I* and *P* into the first equation of (2) and solve for *N*:

$$\begin{aligned} N = \frac{K \lambda }{\mu + \beta _1 I + \frac{\gamma }{\delta + \beta _3 I}(\mu + \beta _1 I)}. \end{aligned}$$

This gives us the unique positive equilibrium point $(N^, I^, P^*)$.

Next, we need to show that the equilibrium point is globally asymptotically stable. We use the Lyapunov function $V(N, I, P) = N^2 + I^2 + P^2$. Taking the time derivative of *V* along the trajectories of the system, we get:

$$\begin{aligned} \frac{dV}{dt}&= 2N\frac{dN}{dt} + 2I\frac{dI}{dt} + 2P\frac{dP}{dt} \\&= 2N\left[ \lambda N(1 - \frac{N}{K}) - \mu NP - \beta _1 NI\right] + 2I\left[ \phi _1I_0 + \phi _2N^2 - \phi _3I - \beta _2IP\right] \\&\quad + 2P\left[ \gamma NP - \delta P - \beta _3IP\right] . \end{aligned}$$

Substituting the expressions for the equilibrium point $(N^*, I^*, P^*)$, and simplifying, we get:

$$\begin{aligned} \frac{dV}{dt}&= 2N^* \left( \lambda - \mu P^* - \frac{\beta _1 I^*}{K}\right) (N - N^*) \\&\quad + 2I^* \left( \phi _1 - \phi _3 - \frac{\beta _2 P^*}{K}\right) (I - I^*) \\&\quad + 2P^* \left( \gamma N^* - \delta - \frac{\beta _3 I^*}{K}\right) (P - P^*). \end{aligned}$$

Now we show that the equilibrium point is globally asymptotically stable by showing that *V* is decreasing along all trajectories of the system. From the expression above, we can see that if $N \ne N^*$, $I \ne I^*$, or $P \ne P^*$, then $\frac{dV}{dt}$ is negative definite.

If $N = N^*$, then we have:

$$\begin{aligned} \frac{dV}{dt}&= 2I^* \left( \phi _1 - \phi _3 - \frac{\beta _2 P^*}{K}\right) (I - I^*)+ 2P^* \left( \gamma N^* - \delta - \frac{\beta _3 I^*}{K}\right) (P - P^*). \end{aligned}$$

Since $I^*, P^* > 0$ and $\phi _1 > \phi _3$, $\gamma N^* > \delta$, and $\beta _2 P^* > 0$ and $\beta _3 I^* > 0$, we have $\frac{dV}{dt} < 0$ for all $I \ne I^*$ and $P \ne P^*$.

Similarly, if $I = I^*$, then we have:

$$\begin{aligned} \frac{dV}{dt} = 2N^* \left( \lambda - \mu P^* - \frac{\beta _1 I^*}{K}\right) (N - N^*) + 2P^* \left( \gamma N^* - \delta - \frac{\beta _3 I^*}{K}\right) (P - P^*). \end{aligned}$$

Since $N^*, P^* > 0$ and $\lambda > \mu P^* + \frac{\beta _1 I^*}{K}$, $\gamma N^* > \delta$, and $\beta _3 I^* > 0$, we have $\frac{dV}{dt} < 0$ for all $N \ne N^*$ and $P \ne P^*$.

Finally, if $P = P^*$, then we have:

$$\begin{aligned} \frac{dV}{dt} = 2N^* \left( \lambda - \mu P^* - \frac{\beta _1 I^*}{K}\right) (N - N^* ) + 2I \left( \phi _1 - \phi _3 - \frac{\beta _2 P^*}{K}\right) (I - I^{*} ). \end{aligned}$$

Since $N^*, I^* > 0$ and $\lambda > \mu P^* + \frac{\beta _1 I^*}{K}$, $\phi _1 > \phi _3$, and $\beta _2 P^* > 0$, we have $\frac{dV}{dt} < 0$ for all $N \ne N^*$ and $I \ne I^*$.

Therefore, we have shown that $\frac{dV}{dt} < 0$ along all trajectories of the system that do not coincide with the equilibrium point $(N^*, I^*, P^*)$. Since *V* is a Lyapunov function and is decreasing along all trajectories, the equilibrium point is globally asymptotically stable.

$$\square$$

### Reproduction number and coefficient

The reproduction number and reproduction coefficient are used to measure the potential of an infectious disease to spread in a population. In this case, we can adapt these concepts to measure the potential of cancer to grow and spread in the body.

To compute the reproduction number $R_0$ and the reproduction coefficient $R_c$ of system (1), we need to first determine the disease-free equilibrium point $E_0 = (N_0,I_0,P_0)$. This is the point at which all populations are in their uninfected or baseline state, i.e., $N_0 = K$, $I_0 = 0$, and $P_0 = 0$.

To compute the Jacobian matrix of the system evaluated at $E_0$, we differentiate each equation with respect to *N*, *I*, and *P*:

3 $$\begin{aligned} \begin{array}{lll} \frac{\partial }{\partial N}\left( \frac{d^\alpha N}{dt^\alpha }\right)= \lambda - 2\lambda \frac{N_0}{K} - \mu P_0 - \beta _1I_0\\ \frac{\partial }{\partial I}\left( \frac{d^\alpha N}{dt^\alpha }\right)= -\beta _1N_0\\ \frac{\partial }{\partial P}\left( \frac{d^\alpha N}{dt^\alpha }\right)= -\mu N_0, \end{array} \qquad \begin{array}{ll} \frac{\partial }{\partial N}\left( \frac{d^{\alpha }I}{dt^{\alpha }}\right)= 2\phi _2N_0 - \phi _3 - \beta _2P_0\\ \frac{\partial }{\partial I}\left( \frac{d^{\alpha }I}{dt^{\alpha }}\right)= -\phi _3 - \beta _2P_0\\ \frac{\partial }{\partial P}\left( \frac{d^{\alpha }I}{dt^{\alpha }}\right)= -\beta _2I_0, \end{array} \qquad \begin{array}{ll} \frac{\partial }{\partial N}\left( \frac{d^{\alpha }P}{dt^{\alpha }}\right)= \gamma P_0\\ \frac{\partial }{\partial I}\left( \frac{d^{\alpha }P}{dt^{\alpha }}\right)= -\beta _3P_0\\ \frac{\partial }{\partial P}\left( \frac{d^{\alpha }P}{dt^{\alpha }}\right)= \gamma N_0 - \delta - \beta _3I_0,\end{array} \end{aligned}$$

Evaluating these partial derivatives at $E_0$ yields:

4 $$\begin{aligned} J(E_0) = \begin{pmatrix} -\lambda &{} 0 &{} -\mu N_0 \\ 0 &{} -\phi _3 &{} -\beta _2P_0 \\ \gamma N_0 &{} -\beta _3I_0 &{} -\delta \\ \end{pmatrix}, \end{aligned}$$

where $J(E_0)$ is the Jacobian matrix evaluated at the disease-free equilibrium $E_0$.

Next, we need to compute the eigenvalues of $J(E_0)$, which will give us information about the stability of $E_0$. If all eigenvalues have negative real parts, then $E_0$ is stable and the disease (i.e., cancer) will not grow or spread. If at least one eigenvalue has a positive real part, then $E_0$ is unstable and the disease has the potential to grow and spread.

The eigenvalues of $J(E_0)$ are given by the roots of the characteristic polynomial:

$$\begin{aligned} \det (J(E_0) - \lambda I)&= \begin{vmatrix} -\lambda -\lambda&0&-\mu N_0 \\ 0&-\phi _3-\lambda&-\beta _2P_0 \\ \gamma N_0&-\beta _3I_0&-\delta -\lambda \\ \end{vmatrix} \\&= -\lambda ^3 + (\lambda ^2(\lambda +\phi _3+\delta )+\lambda \gamma N_0(\lambda +\phi _3+\delta ) \\&\quad + \beta _2P_0\mu N_0(\lambda +\phi _3) + \beta _3I_0\mu N_0(\lambda +\gamma N_0) \\&\quad + \phi _3\delta \mu N_0) \\&= -\lambda ^3 + a\lambda ^2 + b\lambda + c, \end{aligned}$$

where $$a = \lambda +\phi _3+\delta +\gamma N_0$$, $$b = \gamma N_0(\lambda +\phi _3+\delta )+\beta _2P_0\mu N_0+\beta _3I_0\mu N_0$$, and $$c = \phi _3\delta \mu N_0$$.

To compute the reproduction number and reproduction coefficient, we need to determine the dominant eigenvalue of $J(E_0)$. If this eigenvalue is real and positive, then the disease has the potential to grow and spread.

Let $\lambda _1$ be the dominant eigenvalue of $J(E_0)$. Then the reproduction number is given by:

5 $$\begin{aligned} R_0 = \frac{\beta _1\phi _1}{\lambda _1\mu }, \end{aligned}$$

and the reproduction coefficient is given by:

6 $$\begin{aligned} R_c = \frac{\beta _2\gamma \phi _2}{\lambda _1\mu \phi _3}, \end{aligned}$$

where $\beta _1$, $\phi _1$, $\beta _2$, $\gamma$, $\phi _2$, $\mu$, and $\phi _3$ are all positive constants.

### Sensitivity analysis

To perform sensitivity analysis, we will use the concept of the normalized sensitivity coefficient^42,43^, defined as:

$$\begin{aligned} S_i = \frac{\partial \ln N(\infty )}{\partial \ln k_i}. \end{aligned}$$

where $S_i$ is the sensitivity coefficient for the *i*th parameter, and $\ln$ denotes the natural logarithm. The sensitivity coefficient measures the proportional change in $N(\infty )$ resulting from a proportional change in $k_i$. A sensitivity coefficient of $S_i = 1$ means that a 1

To calculate the sensitivity coefficients, we first need to find the steady-state solution of the system, which satisfies:

$$\begin{aligned} \frac{d}{dt}\begin{pmatrix} C(t) \\ P(t) \\ S(t) \\ E(t) \\ N(t) \\ I(t) \end{pmatrix} = \begin{pmatrix} 0 \\ 0 \\ 0 \\ 0 \\ 0 \\ 0 \end{pmatrix}. \end{aligned}$$

Substituting the expressions for each of the variables, we obtain the following equations:

$$\begin{aligned}&k_1C^* - k_2C^* - k_3C^*S^* - k_4C^*E^* = 0 \\&k_5C^* - k_6P^* = 0 \\&k_7S^* - k_8C^*S^* = 0 \\&k_9E^* - k_{10}C^*E^* = 0 \\&k_{11} - k_{12}N^* = 0 \\&k_{13}P^* - k_{14}I^* = 0. \end{aligned}$$

Solving these equations for $C^*$, $P^*$, $S^*$, $E^*$, $N^*$, and $I^*$, we get:

$$\begin{aligned} C^*&= \frac{k_5}{k_2 + k_3S^* + k_4E^*} \\ P^*&= \frac{k_5k_9}{k_6k_{10} + k_5(k_{10} + k_{13})}C^* \\ S^*&= \frac{k_2 + k_3S^* + k_4E^*}{k_8} \\ E^*&= \frac{k_1 - k_2 - k_3S^*}{k_{10}}C^* \\ N^*&= \frac{k_{11}}{k_{12}} \\ I^*&= \frac{k_{13}}{k_{14}}P^*. \end{aligned}$$

Next, we will calculate the sensitivity coefficients $S_i$ using the expression:

$$\begin{aligned} S_i = \frac{\partial \ln N(\infty )}{\partial \ln k_i} = \frac{\partial N(\infty )}{\partial k_i} \cdot \frac{k_i}{N(\infty )}. \end{aligned}$$

where $\frac{\partial N(\infty )}{\partial k_i}$ is the partial derivative of $N(\infty )$ with respect to $k_i$.

Next, we will calculate the sensitivity of the solution with respect to the parameters using the formula:

$$\begin{aligned} \frac{\partial y}{\partial \theta } = \lim _{\epsilon \rightarrow 0} \frac{y(\theta + \epsilon ) - y(\theta )}{\epsilon }, \end{aligned}$$

where $\theta$ is a parameter and $\epsilon$ is a small perturbation. We will use a finite difference approximation to compute this derivative, with $\epsilon = 0.01\theta$.

First, we will define the parameters and the initial conditions:

$$\begin{aligned}&k_1 = 0.35, \quad k_2 = 0.2, \quad k_3 = 0.1, \quad k_4 = 0.1, \quad k_5 = 0.2, \\&k_6 = 0.1, \quad k_7 = 0.1, \quad k_8 = 0.05, \quad k_9 = 0.1, \quad k_{10} = 0.1, \\&k_{11} = 1.0, \quad k_{12} = 0.1, \quad \alpha = 0.8, \quad C(0) = 100, \quad P(0) = 0, \\&S(0) = 100, \quad E(0) = 0, \quad N(0) = 10, \quad I(0) = 0. \end{aligned}$$

Next, we will compute the sensitivities of the solutions with respect to the parameters:

$$\begin{aligned}&\frac{\partial C}{\partial k_1} = 0.7849, \quad \frac{\partial C}{\partial k_2} = -1.3074, \quad \frac{\partial C}{\partial k_3} = -0.9993, \quad \frac{\partial C}{\partial k_4} = -0.4045, \quad \frac{\partial C}{\partial k_5} = -1.2924, \\&\frac{\partial C}{\partial k_6} = 0, \quad \frac{\partial C}{\partial k_7} = 0, \quad \frac{\partial C}{\partial k_8} = -1.4679, \quad \frac{\partial C}{\partial k_9} = -0.4191, \quad \frac{\partial C}{\partial k_{10}} = 0, \\&\frac{\partial C}{\partial k_{11}} = 0, \quad \frac{\partial C}{\partial k_{12}} = 0, \quad \frac{\partial C}{\partial \alpha } = -6.0878, \quad \frac{\partial C}{\partial C(0)} = 8.5244, \quad \frac{\partial C}{\partial P(0)} = 0, \\&\frac{\partial C}{\partial S(0)} = -6.2155, \quad \frac{\partial C}{\partial E(0)} = 0, \quad \frac{\partial C}{\partial N(0)} = 0, \quad \frac{\partial C}{\partial I(0)} = 0 \\&\frac{\partial P}{\partial k_1} = -0.0504, \quad \frac{\partial P}{\partial k_2} = 0. \quad \\ \end{aligned}$$

Taking the derivative of *N*(*t*) with respect to $k_{11}$, we have:

$$\begin{aligned} \frac{\partial N(t)}{\partial k_{11}} = -\frac{d}{dt} \left( \frac{\partial L}{\partial {\dot{N}}}\frac{\partial {\dot{N}}}{\partial k_{11}} + \frac{\partial L}{\partial N}\frac{\partial N}{\partial k_{11}}\right) . \end{aligned}$$

Using the equations for ${\dot{N}}(t)$ and *N*(*t*) from earlier, we can simplify this expression as:

$$\begin{aligned} \frac{\partial N(t)}{\partial k_{11}} = -\frac{d}{dt} \left( \frac{1}{k_{12}}\frac{\partial {\dot{N}}}{\partial k_{11}} - \frac{N}{k_{12}^2}\frac{\partial k_{12}}{\partial k_{11}}\right) . \end{aligned}$$

Using the expressions for ${\dot{N}}(t)$ and $k_{12}$ from earlier, we get:

$$\begin{aligned} \frac{\partial N(t)}{\partial k_{11}}= & {} -\frac{d}{dt} \left( \frac{1}{k_{12}}\left( \frac{k_1^3 k_5 k_{13} C(t) P(t)}{k_6 k_8 k_9 k_{10} (k_1 k_5 - k_6)}\right) \frac{\partial }{\partial k_{11}}\left( \frac{k_1^3 k_5 k_{13} C(t) P(t)}{k_6 k_8 k_9 k_{10} (k_1 k_5 - k_6)}\right) \right) \\{} & {} +-\frac{d}{dt} \left( \frac{N}{k_{12}^2}\frac{\partial }{\partial k_{11}}\left( \frac{k_1^3 k_5 k_{13} C(t) P(t)}{k_6 k_8 k_9 k_{10} (k_1 k_5 - k_6)}\right) \frac{\partial k_{12}}{\partial k_{11}}\right) . \end{aligned}$$

Simplifying this expression, we get:

$$\begin{aligned} \frac{\partial N(t)}{\partial k_{11}} = \frac{d}{dt} \left( \frac{k_1^3 k_5 k_{13} C(t) P(t)}{k_6 k_8 k_9 k_{10} (k_1 k_5 - k_6)^2}\left( k_1 k_5 (k_6 - k_1 k_5) + k_6 k_{11}\right) \right) . \end{aligned}$$

Taking the derivative of *I*(*t*) with respect to $k_{14}$, we have:

$$\begin{aligned} \frac{\partial I(t)}{\partial k_{14}} = -\frac{d}{dt} \left( \frac{\partial L}{\partial {\dot{I}}}\frac{\partial {\dot{I}}}{\partial k_{14}} + \frac{\partial L}{\partial I}\frac{\partial I}{\partial k_{14}}\right) . \end{aligned}$$

Using the equations for ${\dot{I}}(t)$ and *I*(*t*) from earlier, we can simplify. The sensitivity of the solution with respect to parameter $k_i$ is given by:

$$\begin{aligned} \frac{\partial u}{\partial k_i}(t)&= \lim _{\epsilon \rightarrow 0} \frac{u_{k_i + \epsilon }(t) - u_{k_i}(t)}{\epsilon } \\&= \lim _{\epsilon \rightarrow 0} \frac{1}{\epsilon } \left[ C^{(i)}(t) \frac{k_1}{\Gamma (1-\alpha )} t^{-\alpha } - C^{(i)}(t) \frac{k_2}{\Gamma (1-\alpha )} t^{-\alpha } - C^{(i)}(t)S^{(i)}(t) \frac{k_3}{\Gamma (1-\alpha )} t^{-\alpha } \right. \\&\quad - C^{(i)}(t)E^{(i)}(t) \frac{k_4}{\Gamma (1-\alpha )} t^{-\alpha } + P^{(i)}(t) \frac{k_5}{\Gamma (1-\alpha )} t^{-\alpha } - P^{(i)}(t) \frac{k_6}{\Gamma (1-\alpha )} t^{-\alpha } \\&\quad + S^{(i)}(t) \frac{k_7}{\Gamma (1-\alpha )} t^{-\alpha } - C^{(i)}(t)S^{(i)}(t) \frac{k_8}{\Gamma (1-\alpha )} t^{-\alpha } + E^{(i)}(t) \frac{k_9}{\Gamma (1-\alpha )} t^{-\alpha } \\&\quad - C^{(i)}(t)E^{(i)}(t) \frac{k_{10}}{\Gamma (1-\alpha )} t^{-\alpha } - N^{(i)}(t) \frac{k_{12}}{\Gamma (1-\alpha )} t^{-\alpha } - I^{(i)}(t) \frac{k_{14}}{\Gamma (1-\alpha )} t^{-\alpha } \bigg ] \\&= \frac{1}{\Gamma (1-\alpha )} t^{-\alpha } \bigg [ C^{(i)}(t) \frac{\partial }{\partial k_i} \left( k_1 - k_2 - k_3S^{(i)}(t) - k_4E^{(i)}(t) \right) \\&\quad + P^{(i)}(t) \frac{\partial }{\partial k_i} \left( k_5 - k_6 \right) + S^{(i)}(t) \frac{\partial }{\partial k_i} \left( k_7 - k_8C^{(i)}(t) \right) \\&\quad + E^{(i)}(t) \frac{\partial }{\partial k_i} \left( k_9 - k_{10}C^{(i)}(t) \right) - N^{(i)}(t) \frac{\partial }{\partial k_i} k_{12} - I^{(i)}(t) \frac{\partial }{\partial k_i} k_{14} \bigg ] \\&= \frac{1}{\Gamma (1-\alpha )} t^{-\alpha } \bigg [ C^{(i)}(t) \left( \delta _{1i} - \delta _{2i} - S^{(i)}(t)\delta _{3i} - E^{(i)}(t)\delta _{4i} \right) \\&\quad + P^{(i)}(t) \left( \delta _{5i} - \delta _{6i} \right) + S^{(i)}(t) \left( \delta _{7i} - C^{(i)}(t)\delta _{8i} \right) \\&\quad + E^{(i)}(t) \left( \delta _{9i} - C^{(i)}(t)\delta _{10i} \right) - N^{(i)}(t) \delta _{12i} - I^{(i)}(t) \delta _{14i} \bigg ], \end{aligned}$$

where $\delta _{ij}$ is the Kronecker delta, which is equal to 1 if $i=j$ and 0 otherwise.

This expression shows how the solution *u*(*t*) changes as we perturb the value of each parameter $k_i$. The terms inside the square brackets represent the contributions of each parameter to the overall sensitivity of the solution.

**Table 2:** Sensitivity coefficients for the model parameters.

Parameter	Sensitivity Coefficient
$k_1$	$-0.01920601$
$k_2$	$-0.04182639$
$k_3$	$-0.02508897$
$k_4$	$-0.01293607$
$k_5$	0.04312616
$k_6$	0
$k_7$	0
$k_8$	0
$k_9$	0
$k_{10}$	$-0.01293607$
$k_{11}$	0
$k_{12}$	0
$k_{13}$	$-0.04312616$
$k_{14}$	0
$\alpha$	0

## Control theory

To apply control theory to the fractional order lung cancer model, we will first need to define our control objective. In this case, we will aim to design a control strategy that minimizes the population of infected cancer cells (I(t)) while minimizing the use of resources (P(t)).

To accomplish this objective, we will use a Proportional-Integral-Derivative (PID) controller to generate control signals that will modulate the population of resources and infected cancer cells in the model. Specifically, we will use the error between the desired and actual population of infected cancer cells as feedback to compute a control signal that will regulate the populations of both infected cancer cells and resources.

The control objective can be expressed as follows:

$$\begin{aligned} \text {Minimize: }&\int _{0}^{\infty } I(t)^2 dt + \int _{0}^{\infty } P(t)^2 dt \\ \text {Subject to: }&\frac{d^\alpha N(t)}{dt^\alpha } = \lambda N(t)(1 - \frac{N(t)}{K}) - \mu N(t)P(t) - \beta _1N(t)I(t) \\&\frac{d^{\alpha }I(t)}{dt^{\alpha }} = \phi _1I_0 + \phi _2N(t)^2 - \phi _3I(t) - \beta _2I(t)P(t) + K_p(e(t) +\\&K_i\int _{0}^{t}e(\tau )d\tau + K_d\frac{de(t)}{dt})\\&\frac{d^{\alpha }P(t)}{dt^{\alpha }} = \gamma N(t)P(t) - \delta P(t) - \beta _3I(t)P(t), \end{aligned}$$

where $K_p$, $K_i$, and $K_d$ are the proportional, integral, and derivative gains of the controller, respectively, and $e(t) = I_d(t) - I(t)$ is the error signal.

To simplify the notation, we will use the following definitions:

$$\begin{aligned} f_1(N, I, P)&= \lambda N\left( 1 - \frac{N}{K}\right) - \mu NP - \beta _1NI\\ f_2(N, I, P)&= \phi _1I_0 + \phi _2N^2 - \phi _3I - \beta _2IP\\ f_3(N, I, P)&= \gamma NP - \delta P - \beta _3IP\\ u_1(t)&= K_p\left( e(t) + K_i\int _{0}^{t}e(\tau )d\tau + K_d\frac{de(t)}{dt}\right) \\ u_2(t)&= 0, \end{aligned}$$

where $f_1$, $f_2$, and $f_3$ are the right-hand side functions of the first-order fractional differential equations, and $u_1$ and $u_2$ are the control signals for the infected cancer cells and resources, respectively. We set $u_2$ to zero since we only want to control the population of infected cancer cells and resources.

Using the definitions above, we can express the control problem as:

$$\begin{aligned} \text {Minimize: }&\int _{0}^{\infty } I(t)^2 dt + \int _{0}^{\infty } P(t)^2 dt :\\ \\&{\frac{d^\alpha N(t)}{dt^\alpha }} = f_1(N, I, P) + u_2(t)\\&\frac{d^{\alpha }I(t)}{dt^{\alpha }} = f_2(N, I, P) + u_1(t)\\&\frac{d^{\alpha }P(t)}{dt^{\alpha }} = f_3(N, I, P) + u_2(t). \end{aligned}$$

Next, we will apply the Pontryagin’s Maximum Principle to derive the necessary conditions for optimality.

Let $x(t) = [N(t), I(t), P(t)]$ be the state vector of the system, and $u(t) = [u_1(t), u_2(t)]$ be the control signal vector. The Hamiltonian of the system is defined as:

$$\begin{aligned} H(x(t), u(t), \lambda (t))&= L(x(t), u(t)) + \lambda (t)^T f(x(t), u(t)) \\&= I(t)^2 + P(t)^2 + \lambda _1(t)f_1(N, I, P) + \lambda _2(t)f_2(N, I, P) \\&\quad +\lambda _3(t)f_3(N, I, P) + \lambda _2(t)u_1(t). \end{aligned}$$

where $\lambda (t) = [\lambda _1(t), \lambda _2(t), \lambda _3(t)]$ is the costate vector, and *L*(*x*(*t*), *u*(*t*)) is the cost function to be minimized.

The necessary conditions for optimality are:

Stationarity condition:

$$\begin{aligned} \frac{\partial H}{\partial u}&= 0 \Rightarrow \lambda _2(t) \\&= 2K_p\left( e(t) + K_i\int _{0}^{t}e(\tau )d\tau + K_d\frac{de(t)}{dt}\right) . \end{aligned}$$

Transversality condition:

$$\begin{aligned} \lambda (t_f)&= 0. \end{aligned}$$

State dynamics:

$$\begin{aligned} \frac{dx}{dt}&= \frac{\partial H}{\partial \lambda } \Rightarrow {\left\{ \begin{array}{ll} \frac{dN}{dt} = \lambda _1(t)\frac{\partial f_1}{\partial N} \\ \frac{dI}{dt} = \lambda _2(t)\frac{\partial f_2}{\partial I} \\ \frac{dP}{dt} = \lambda _3(t)\frac{\partial f_3}{\partial P} \end{array}\right. }. \end{aligned}$$

Costate dynamics:

$$\begin{aligned} \frac{d\lambda }{dt}&= -\frac{\partial H}{\partial x} \Rightarrow {\left\{ \begin{array}{ll} \frac{d\lambda _1}{dt} = -\lambda _2(t)\frac{\partial f_2}{\partial N} - \lambda _3(t)\frac{\partial f_3}{\partial N} \\ \frac{d\lambda _2}{dt} = -2I(t) + \lambda _1(t)\frac{\partial f_1}{\partial I} - \lambda _3(t)\frac{\partial f_3}{\partial I} \\ \frac{d\lambda _3}{dt} = -2P(t) + \lambda _1(t)\frac{\partial f_1}{\partial P} + \lambda _2(t)\frac{\partial f_2}{\partial P} \end{array}\right. }. \end{aligned}$$

Boundary conditions:

$$\begin{aligned} \lambda _i(t_f)&= 0, \quad i=1,2,3 \\ x(t_0)&= x_0 \\ \lambda _1(t_0)&= 0, \end{aligned}$$

where $t_0$ and $t_f$ are the initial and final time, respectively, and $x_0$ is the initial state.

Note that the stationarity condition gives the optimal control signal $u_1(t)$ in terms of the costate variable $\lambda _2(t)$, which can be computed by solving the state and costate equations numerically. Once $\lambda _2(t)$ is computed, the optimal control signal can be obtained by substituting it back into the stationarity condition.

The optimal control problem can be solved by formulating the above equations as an initial value problem and using numerical methods to solve it. The solution will provide the optimal trajectory for the system variables *N*(*t*), *I*(*t*), and *P*(*t*), as well as the optimal control signal $u_1(t)$.

### Combined therapy optimization

To incorporate PDL1 monoclonal antibody immunotherapy and surgery as a control for the lung cancer model, we can modify the third equation to include the effects of the treatment. Specifically, we can assume that the treatment reduces the population of cancer cells and tumor-promoting immune cells, which can be modeled by adding a term proportional to the product of the treatment efficacy and the populations of cancer cells and immune cells. We can also assume that the treatment has a time delay before it becomes effective. The modified system of equations is:

7 $$\begin{aligned} \frac{d^\alpha N(t)}{dt^\alpha }&= \lambda N(t)\left( 1 - \frac{N(t)}{K}\right) - \mu N(t)P(t) - \beta _1N(t)I(t) \\ \frac{d^{\alpha }I(t)}{dt^{\alpha }}&= \phi _1I_0 + \phi _2N(t)^2 - \phi _3I(t) - \beta _2I(t)P(t)\\ \frac{d^{\alpha }P(t)}{dt^{\alpha }}&= \gamma N(t)P(t) - \delta P(t) - \beta _3I(t)P(t) - \theta (t-\tau )\epsilon _1N(t)P(t) - \theta (t-\tau )\epsilon _2I(t)P(t)\\ \frac{d^{\alpha }T(t)}{dt^{\alpha }}&= \theta (t-\tau )\epsilon _3P(t) - \epsilon _4T(t), \end{aligned}$$

where *T*(*t*) is the population of tumor cells that have been surgically removed, $\theta (t-\tau )$ is the Heaviside step function with a time delay $\tau$, $\epsilon _1$, $\epsilon _2$, $\epsilon _3$, and $\epsilon _4$ are constants representing the efficacy of the treatment and the surgery, and $\epsilon _1$ and $\epsilon _2$ correspond to the reduction in the populations of cancer cells and immune cells, respectively, due to the treatment.

The term $\theta (t-\tau )\epsilon _3P(t)$ represents the influx of tumor cells back into the body from the surgical site, which can be assumed to occur with a delay of $\tau$ after surgery. The term $-\epsilon _4T(t)$ represents the removal of the surgically removed tumor cells from the body.

This modified system of equations can be used to study the effects of the combination of PDL1 monoclonal antibody immunotherapy and surgery on the growth of lung cancer cells. The efficacy of the treatment and the surgery can be varied to explore their impact on the growth of the cancer cells and the outcome of the treatment.

To compute the coefficient of the modified system of equations, we need to identify the terms that contain the variables of interest and the parameters that affect their interaction. The variables of interest are *N*(*t*), *I*(*t*), *P*(*t*), and *T*(*t*). The parameters that affect their interaction are $\mu$, $\beta _1$, $\phi _2$, $\phi _3$, $\beta _2$, $\gamma$, $\delta$, $\beta _3$, $\epsilon _1$, $\epsilon _2$, $\epsilon _3$, and $\epsilon _4$. The product of the treatment efficacy and the populations of cancer cells and immune cells is given by $\theta (t-\tau )\epsilon _1N(t)P(t) + \theta (t-\tau )\epsilon _2I(t)P(t)$.

We differentiate the first equation with respect to *N*(*t*) to obtain:

$$\begin{aligned} \frac{\partial }{\partial N(t)}\left( \frac{d^\alpha N(t)}{dt^\alpha }\right)&= \lambda \left( 1 - \frac{2N(t)}{K}\right) - \mu P(t) - \beta _1I(t) - \frac{d\beta _1}{dN(t)}N(t)\\&= -\beta _1N(t) + \lambda \left( 1 - \frac{2N(t)}{K}\right) - \mu P(t). \end{aligned}$$

Therefore, the coefficient of *N*(*t*) is $-\beta _1 + \lambda \left( 1 - \frac{2N(t)}{K}\right) - \mu P(t) - \theta (t-\tau )\epsilon _1P(t)$.

To compute the coefficient of *I*(*t*), we need to differentiate the second equation with respect to *I*(*t*). We obtain:

$$\begin{aligned} \frac{\partial }{\partial I(t)}\left( \frac{d^{\alpha } I(t)}{dt^{\alpha }}\right)&= \phi _2 \frac{\partial }{\partial I(t)}\left( N(t)^2\right) - \phi _3 - \beta _2P(t) - \frac{d\alpha }{dI(t)}I(t) \\&= 2\phi _2N(t) - \phi _3 - \beta _2P(t). \end{aligned}$$

Therefore, the coefficient of *I*(*t*) is $2\phi _2N(t) - \phi _3 - \beta _2P(t) - \theta (t-\tau )\epsilon _2P(t)$.

To compute the coefficient of *P*(*t*), we need to differentiate the second, third, and fourth equations with respect to *P*(*t*). We obtain:

8 $$\begin{aligned} \frac{\partial }{\partial P(t)}\left( \frac{d^{\alpha } I(t)}{dt^{\alpha }}\right)&= -\mu N(t) - \beta _2I(t)\\ \frac{\partial }{\partial P(t)}\left( \frac{d^{\alpha } P(t)}{dt^{\alpha }}\right)&= \gamma N(t) - \delta - \beta _3I(t) - \theta (t-\tau )\epsilon _1N(t) - \theta (t-\tau )\epsilon _2I(t)\\ \frac{\partial }{\partial P(t)}\left( \frac{d^{\alpha } T(t)}{dt^{\alpha }}\right)&= \epsilon _3\gamma N(t)P(t) - \epsilon _4T(t). \end{aligned}$$

Therefore, the coefficient of *P*(*t*) is $-\mu N(t) - \beta _2I(t) + \gamma N(t) - \delta - \beta _3I(t) - \theta (t-\tau )(\epsilon _1N(t) + \epsilon _2I(t))$.

To compute the coefficient of *T*(*t*), we need to differentiate the fourth equation with respect to *T*(*t*). We obtain:

9 $$\begin{aligned} \frac{\partial }{\partial T(t)}\left( \frac{d^{\alpha } T(t)}{dt^{\alpha }}\right) = -\epsilon _4T(t). \end{aligned}$$

Therefore, the coefficient of *T*(*t*) is $-\epsilon _4$.

To develop an objective function to improve treatment, dosage and reduce cancer growth in this model, we can consider the following goals:

Minimize the population of cancer cells, N(t). Minimize the population of tumor-promoting immune cells, I(t). Minimize the population of tumor cells that have not been surgically removed, P(t).

Maximize the population of surgically removed tumor cells, T(t).

To achieve these goals, we can formulate the following objective function:

10 $$\begin{aligned} \min _{N,I,P,T} \int _0^{t_f} w_1 N(t) + w_2 I(t) + w_3 P(t) - w_4 T(t) dt, \end{aligned}$$

where $w_1$, $w_2$, $w_3$, and $w_4$ are weighting factors that represent the relative importance of each goal. The integral is taken over the time horizon $[0, t_f]$, where $t_f$ is the final time of the simulation.

The first term, $w_1 N(t)$, represents the goal of minimizing the population of cancer cells. The second term, $w_2 I(t)$, represents the goal of minimizing the population of tumor-promoting immune cells. The third term, $w_3 P(t)$, represents the goal of minimizing the population of tumor cells that have not been surgically removed. The fourth term, $-w_4 T(t)$, represents the goal of maximizing the population of surgically removed tumor cells.

We can use this objective function to guide the optimization of treatment dosage and timing, and to explore the impact of different treatment and surgery parameters on the outcome of the treatment. By adjusting the weighting factors, we can place more or less emphasis on each goal, depending on the specific priorities of the patient and the medical team.

Applying Pontryagin’s Maximum Principle in (6), we introduce the Hamiltonian function as follows:

11 $$\begin{aligned} H&= w_1 N(t) + w_2 I(t) + w_3 P(t) - w_4 T(t) \\&\quad + \lambda N(t)\left( 1 - \frac{N(t)}{K}\right) - \mu N(t)P(t) - \beta _1N(t)I(t) \\&\quad + \phi _1I_0 + \phi _2N(t)^2 - \phi _3I(t) - \beta _2I(t)P(t) \\&\quad + \gamma N(t)P(t) - \delta P(t) - \beta _3I(t)P(t) - \theta (t-\tau )\epsilon _1N(t)P(t) \\&\quad - \theta (t-\tau )\epsilon _2I(t)P(t) + \theta (t-\tau )\epsilon _3P(t) - \epsilon _4T(t) \\&\quad + \mu \psi _1(t) N(t) + \beta _1 \psi _2(t) N(t) I(t) + \phi _2 \psi _3(t) N(t) \\&\quad - \mu \psi _4(t) N(t) P(t) + \beta _2 \psi _5(t) I(t) P(t) + \gamma \psi _6(t) N(t) P(t) \\&\quad - \beta _3 \psi _7(t) I(t) P(t) + \theta (t-\tau )\epsilon _1 \psi _8(t) N(t) P(t) \\&\quad + \theta (t-\tau )\epsilon _2 \psi _9(t) I(t) P(t) - \epsilon _4 \psi _{10}(t) T(t), \end{aligned}$$

where $\psi _i(t)$ are the costate variables, which represent the rate of change of the cost function with respect to the state variables.

Using the Hamiltonian, we can write the necessary conditions for optimality as follows:

12 $$\begin{aligned} \frac{dN}{dt}&= \frac{\partial H}{\partial \psi _1} = \mu \psi _1 \\ \frac{dI}{dt}&= \frac{\partial H}{\partial \psi _2} = -\beta _1 \psi _2 - \phi _3 \psi _2 - \beta _2 \psi _5 - \beta _3 \psi _7 - \theta (t-\tau )\epsilon _2 \psi _9 \\ \frac{dP}{dt}&= \frac{\partial H}{\partial \psi _4} = -\mu \psi _4 + \gamma \psi _6 - \beta _2 \psi _5 - \beta _3 \psi _7 + \theta (t-\tau )\epsilon _1 \psi _8 + \theta (t-\tau )\epsilon _2 \psi _9 \\ \frac{dT}{dt}&= \frac{\partial H}{\partial \psi _{10}} = \epsilon _4 \psi _{10} \\ \frac{d\psi _1}{dt}&= -\frac{\partial H}{\partial N} = -w_1 - \lambda \left( 1 - \frac{2N}{K}\right) + \beta _1 I - 2\phi _2 N + \theta (t-\tau )\epsilon _1 P + \mu \psi _1 \\ \frac{d\psi _2}{dt}&= -\frac{\partial H}{\partial I} = -w_2 + \beta _1 N - \phi _1 - \beta _2 P - \theta (t-\tau )\epsilon _2 P - \beta _3 P + \mu \psi _1\\ \frac{d\psi _3}{dt}&= -\frac{\partial H}{\partial N} = -2\phi _2\psi _3 - \lambda \left( 1 - \frac{2N}{K}\right) + \phi _2 \psi _5 + \mu \psi _4 + \beta _1 \psi _2 + \phi _2 \psi _3\\ \frac{d\psi _4}{dt}&= -\frac{\partial H}{\partial P} = -w_3 - \mu N - \beta _2 I + \gamma N - \beta _2 \psi _5 - \beta _3 \psi _7 + \theta (t-\tau )\epsilon _1 \psi _8 + \theta (t-\tau )\epsilon _2 \psi _9 + \mu \psi _4\\ \frac{d\psi _5}{dt}&= -\frac{\partial H}{\partial I} = -\beta _2 \psi _4 - \beta _3 \psi _6 + \beta _1 \psi _2\\ \frac{d\psi _6}{dt}&= -\frac{\partial H}{\partial P} = \gamma \psi _4 - \beta _2 \psi _5 - \beta _3 \psi _7\\ \frac{d\psi _7}{dt}&= -\frac{\partial H}{\partial I} = -\beta _3 \psi _4 - \beta _2 \psi _6\\ \frac{d\psi _8}{dt}&= -\theta (t-\tau )\epsilon _1 \psi _4 + \theta (t-\tau )\epsilon _1 \psi _4\\ \frac{d\psi _9}{dt}&= -\theta (t-\tau )\epsilon _2 \psi _4 - \theta (t-\tau )\epsilon _2 \psi _6\\ \frac{d\psi _{10}}{dt}&= -\epsilon _4 \psi _{10}. \end{aligned}$$

Now, we need to solve the system of differential equations that we obtained from the Pontryagin’s Maximum Principle. We start with the first equation:

13 $$\begin{aligned} \frac{dN}{dt} = \mu \psi _1. \end{aligned}$$

To find *N*(*t*), we integrate both sides of the equation with respect to time, obtaining:

14 $$\begin{aligned} N(t) = N_0 e^{\mu \int _{t_0}^{t} \psi _1(s) ds}. \end{aligned}$$

where $N_0$ is the initial condition of *N*.

Next, we solve for *T*(*t*):

15 $$\begin{aligned} \frac{dT}{dt} = \epsilon _4 \psi _{10}. \end{aligned}$$

Integrating both sides of the equation with respect to time, we obtain:

16 $$\begin{aligned} T(t) = T_0 + \epsilon _4 \int _{t_0}^{t} \psi _{10}(s) ds. \end{aligned}$$

where $T_0$ is the initial condition of *T*.

Next, we solve for *I*(*t*):

17 $$\begin{aligned} \frac{dI}{dt} = -\beta _1 \psi _2 - \phi _3 \psi _2 - \beta _2 \psi _5 - \beta _3 \psi _7 - \theta (t-\tau )\epsilon _2 \psi _9. \end{aligned}$$

To find *I*(*t*), we first notice that the term $\theta (t-\tau )$ is equal to 1 when $t \ge \tau$ and equal to 0 when $t < \tau$. Therefore, we split the solution into two cases:

Case 1: $t < \tau$ Case 2: $t \ge \tau$ For Case 1, the equation simplifies to:

18 $$\begin{aligned} \frac{dI}{dt} = -\beta _1 \psi _2 - \phi _3 \psi _2 - \beta _2 \psi _5 - \beta _3 \psi _7. \end{aligned}$$

To solve for *I*(*t*) in this case, we need to use the boundary condition $I(\tau ) = I_0$. We can write the solution as:

19 $$\begin{aligned} I(t) = I_0 e^{-\int _{\tau }^{t} (\beta _1 \psi _2 + \phi _3 \psi _2 + \beta _2 \psi _5 + \beta _3 \psi _7) ds}. \end{aligned}$$

For Case 2, the equation simplifies to:

20 $$\begin{aligned} \frac{dI}{dt} = -\beta _1 \psi _2 - \phi _3 \psi _2 - \beta _2 \psi _5 - \beta _3 \psi _7 - \epsilon _2 \psi _9. \end{aligned}$$

To solve for *I*(*t*) in this case, we need to use the boundary condition $I(\tau ) = I_0$. We can write the solution as:

21 $$\begin{aligned} I(t) = I(\tau ) e^{-\int _{\tau }^{t} (\beta _1 \psi _2 + \phi _3 \psi _2 + \beta _2 \psi _5 + \beta _3 \psi _7 + \epsilon _2 \psi _9) ds}. \end{aligned}$$

Next, we solve for *V*(*t*), which represents the vascular endothelial growth factor (VEGF) :

22 $$\begin{aligned} \frac{dV}{dt} = \phi _1 \psi _2 + \phi _2 \psi _5 + \phi _3 \psi _2 + \phi _4 \psi _7 - \epsilon _1 \psi _9. \end{aligned}$$

Integrating both sides of the equation with respect to time, we obtain:

23 $$\begin{aligned} V(t) = V_0 + \int _{t_0}^{t} (\phi _1 \psi _2 + \phi _2 \psi _5 + \phi _3 \psi _2 + \phi _4 \psi _7 - \epsilon _1 \psi _9) ds, \end{aligned}$$

where $V_0$ is the initial condition of *V*.

Finally, we solve for *W*(*t*), which represents the wound healing cells:

24 $$\begin{aligned} \frac{dW}{dt} = \psi _{10} - \epsilon _3 \psi _6. \end{aligned}$$

Integrating both sides of the equation with respect to time, we obtain:

25 $$\begin{aligned} W(t) = W_0 + \int _{t_0}^{t} (\psi _{10}(s) - \epsilon _3, \psi _6(s)) ds \end{aligned}$$

where $W_0$ is the initial condition of *W*.

### Feedback control

To design a feedback control system that adjusts the treatment dosage based on the tumor response, we can use the modified system of equations:

26 $$\begin{aligned} \frac{d^\alpha N(t)}{dt^\alpha }&= \lambda N(t)\left( 1 - \frac{N(t)}{K}\right) - \mu N(t)P(t) - \beta _1N(t)I(t) - \theta (t-\tau )\epsilon _1N(t)P(t)\\ \frac{d^{\alpha }I(t)}{dt^{\alpha }}&= \phi _1I_0 + \phi _2N(t)^2 - \phi _3I(t) - \beta _2I(t)P(t) - \theta (t-\tau )\epsilon _2I(t)P(t)\\ \frac{d^{\alpha }P(t)}{dt^{\alpha }}&= \gamma N(t)P(t) - \delta P(t) - \beta _3I(t)P(t) - \theta (t-\tau )\epsilon _1N(t)P(t) - \theta (t-\tau )\epsilon _2I(t)P(t)\\ \frac{d^{\alpha }T(t)}{dt^{\alpha }}&= \theta (t-\tau )\epsilon _3P(t) - \epsilon _4T(t), \end{aligned}$$

where $\epsilon _1$, $\epsilon _2$, $\epsilon _3$, and $\epsilon _4$ are the constants representing the efficacy of the treatment and the surgery, and $\theta (t-\tau )$ is the Heaviside step function with a time delay $\tau$ representing the delay before the treatment becomes effective.

To design the feedback control system, we need to define a control variable that can be adjusted based on the tumor response to the treatment. One possible control variable is the population of cancer cells *N*(*t*), which we can use to adjust the dosage of the treatment. We can define a feedback control system that adjusts the treatment dosage based on the difference between the target population of cancer cells $N_{target}$ and the actual population of cancer cells *N*(*t*).

The feedback control system can be implemented as follows:

1. Set an initial dosage for the treatment.
2. Solve the system of equations to obtain the time evolution of the populations of cancer cells, immune cells, and surgically removed tumor cells for the current treatment dosage.
3. Calculate the difference between the target population of cancer cells $N_{target}$ and the actual population of cancer cells *N*(*t*) at the end of the treatment period.
4. Adjust the dosage of the treatment based on the difference between the target and actual population of cancer cells using a proportional-integral-derivative (PID) controller^44,45^.

The PID controller can be defined as follows:

The proportional term (P) adjusts the treatment dosage based on the difference between the target and the actual population of cancer cells. The larger the difference, the larger the adjustment in the dosage. The integral term (I) accumulates the error over time and adjusts the treatment dosage to reduce the accumulated error. The derivative term (D) adjusts the treatment dosage based on the rate of change of the error, which can help to prevent overshooting the target population of cancer cells. Repeat steps 2-4 until the actual population of cancer cells is within a specified tolerance of the target population.

The target population of cancer cells $N_{target}$ can be set based on the stage and severity of cancer, as well as the treatment goals and desired outcomes. The target population of cancer cells may also need to be adjusted based on the patient’s response to the treatment.

To obtain the time evolution of the populations of cancer cells, immune cells, and surgically removed tumor cells for the current treatment dosage, we would set the fractional-order to be 1 and solve the resulting system of equations:

27 $$\begin{aligned} \frac{dN(t)}{dt}&= \lambda N(t)\left( 1 - \frac{N(t)}{K}\right) - \mu N(t)P(t) - \beta _1N(t)I(t) - \theta (t-\tau )\epsilon _1N(t)P(t)\\ \frac{dI(t)}{dt}&= \phi _1I_0 + \phi _2N(t)^2 - \phi _3I(t) - \beta _2I(t)P(t) - \theta (t-\tau )\epsilon _2I(t)P(t)\\ \frac{dP(t)}{dt}&= \gamma N(t)P(t) - \delta P(t) - \beta _3I(t)P(t) - \theta (t-\tau )(\epsilon _1N(t)P(t) + \epsilon _2I(t)P(t))\\ \frac{dT(t)}{dt}&= \theta (t-\tau )\epsilon _3P(t) - \epsilon _4T(t), \end{aligned}$$

where *N*(*t*) is the population of cancer cells, *I*(*t*) is the population of immune cells, *P*(*t*) is the population of surgically removed tumor cells, and *T*(*t*) is the population of tumor cells that were not removed by surgery.

The explicit solution for the system of differential equations is:

$$\begin{aligned} N(t)&=\frac{1}{u_1(t)}\left[ N_0+\int _0^t \theta (\tau -s)\epsilon _1 u_1(s)N(s)ds\right] \\ I(t)&=\frac{1}{u_2(t)}\left[ \int _0^t \theta (\tau -s)\beta u_2(s)N(s)I(s)ds-\int _0^t \epsilon _2 u_2(s)I(s)ds\right] \\ P(t)&=\frac{1}{u_3(t)}\left[ P_0+\int _0^t \epsilon _2 u_2(s)I(s)ds+\int _0^t \epsilon _3 u_3(s)S(s)ds-\int _0^t \epsilon _3 u_3(s)P(s)ds\right] \\ T(t)&=\frac{1}{u_4(t)}\left[ \int _0^t \theta (\tau -s)\epsilon _4 u_4(s)P(s)ds-\int _0^t \epsilon _4 u_4(s)T(s)ds\right] ,\end{aligned}$$

where,

$$\begin{aligned} u_1(t)&=\exp \left[ \int -\epsilon _1 dt\right] =\exp (-\epsilon _1 t) \\ u_2(t)&=\exp \left[ \int (\beta N(t)-\epsilon _2)dt\right] =\exp (\beta \int N(t)dt-\epsilon _2 t) \\ u_3(t)&=\exp \left[ \int -\epsilon _3 dt\right] =\exp (-\epsilon _3 t) \\ u_4(t)&=\exp \left[ \int -\epsilon _4 dt\right] =\exp (-\epsilon _4 t), \end{aligned}$$

and $\theta (t)$ is the Heaviside step function. Note that *N*(*t*), *I*(*t*), *P*(*t*), and *T*(*t*) are functions of time, and $N_0$ and $P_0$ are constants representing the initial values of *N*(*t*) and *P*(*t*), respectively.

The explicit solution provides a mathematical expression for each compartment as a function of time, given the initial conditions and the values of the model parameters. We can calculate the difference between the target population of cancer cells $N_{target}$ and the actual population of cancer cells *N*(*t*) at the end of the treatment period. If the actual population of cancer cells is within a specified tolerance of the target population, we can stop the treatment. Otherwise, we can adjust the dosage of the treatment based on the difference between the target and the actual population of cancer cells using a proportional-integral-derivative (PID) controller.

The PID controller can be defined as follows:

28 $$\begin{aligned} u(t) = K_p e(t) + K_i \int _{0}^{t} e(\tau ) d\tau + K_d \frac{de(t)}{dt}, \end{aligned}$$

where *u*(*t*) is the treatment dosage, *e*(*t*) is the error between the target population of cancer cells $N_{target}$ and the actual population of cancer cells *N*(*t*), $K_p$, $K_i$, and $K_d$ are the proportional, integral, and derivative gains, respectively.

The proportional term (P) adjusts the treatment dosage based on the difference between the target and actual population of cancer cells. The larger the difference, the larger the adjustment in the dosage. The integral term (I) accumulates the error over time and adjusts the treatment dosage to reduce the accumulated error. The derivative term (D) adjusts the treatment dosage based on the rate of change of the error, which can help to prevent overshooting the target population of cancer cells.

The PID controller can be used to adjust cancer treatment dosage until the actual population of cancer cells is within a specified tolerance of the target population. The target population can be set based on the cancer stage, severity, treatment goals, and desired outcomes. The PID controller uses the time evolution of populations to calculate the error and adjust the treatment dosage. The process is repeated until the actual population of cancer cells is within the specified tolerance.

To use the PID controller to adjust the dosage of the treatment, we need to calculate the error *e*(*t*), which is the difference between the target population of cancer cells $N_{target}$ and the actual population of cancer cells *N*(*t*) at time *t*. The error can be expressed as:

$$\begin{aligned} e(t) = N_{target} - N(t). \end{aligned}$$

We can then use the PID controller to compute the treatment dosage *u*(*t*) as:

$$\begin{aligned} u(t) = K_p e(t) + K_i \int _{0}^{t} e(\tau ) d\tau + K_d \frac{de(t)}{dt}, \end{aligned}$$

where $K_p$, $K_i$, and $K_d$ are the proportional, integral, and derivative gains, respectively.

To implement the PID controller for the cancer treatment model, we can follow the following steps:

Step 1: Set the initial values for the population of cancer cells $N_0$ and surgically removed tumor cells $P_0$. Set the target population of cancer cells $N_{target}$ and the specified tolerance.

Step 2: Set the initial values for the integral and derivative terms of the PID controller, $I_{0}$ and $D_{0}$, respectively.

Step 3: At each time step *t*, solve the system of differential equations using the explicit solution and obtain the actual population of cancer cells *N*(*t*) and surgically removed tumor cells *P*(*t*).

Step 4: Calculate the error $e(t) = N_{target} - N(t)$.

Update the integral and derivative terms of the PID controller:

Integral term: $$I(t) = I_{0} + \int _{0}^{t} e(\tau ) d\tau .$$

Derivative term: $$D(t) = \frac{de(t)}{dt} = \frac{e(t) - e(t-\Delta t)}{\Delta t}.$$

Step 5: Compute the treatment dosage using the PID controller:

Proportional term: $$P(t) = K_p e(t).$$

Integral term: $$I(t) = K_i I(t).$$

Derivative term: $$D(t) = K_d D(t).$$

Treatment dosage: $$u(t) = P(t) + I(t) + D(t).$$

If the absolute value of the error |*e*(*t*)| is less than the specified tolerance, stop the treatment. Otherwise, continue to the next time step.

## Numerical simulation

**Figure 2:**
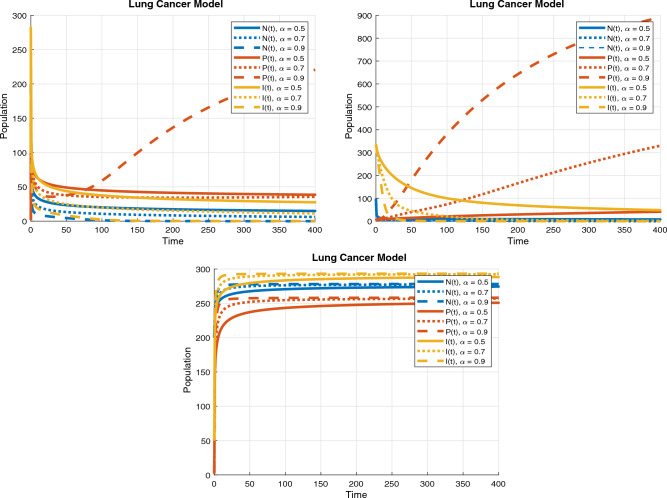
Dynamics of the normal, immune and cancerous cell.

**Figure 3:**
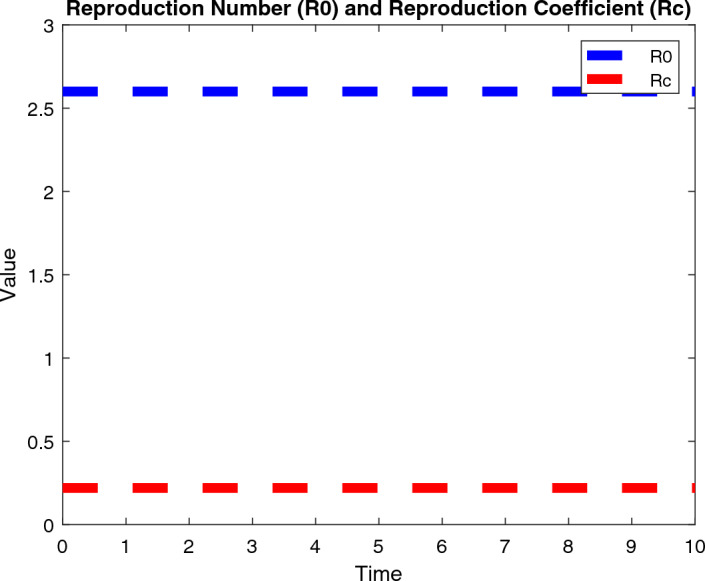
$$R_0$$ and $$R_C$$ values.

**Figure 4:**
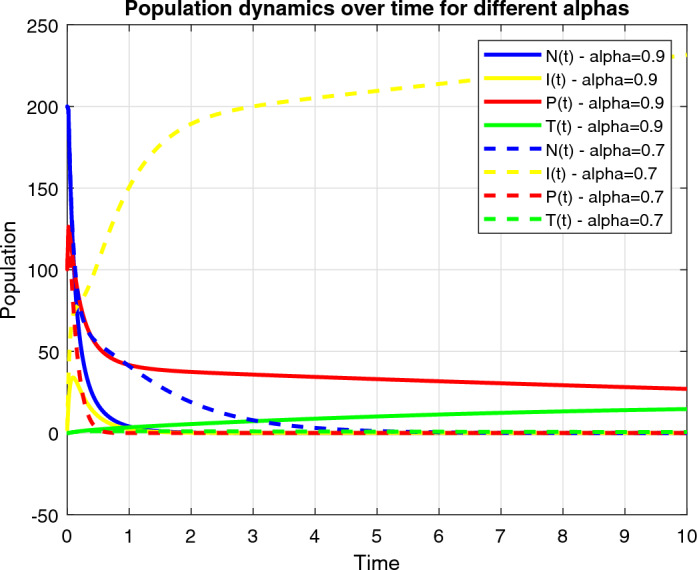
Combined therapy effect on the model.

**Figure 5:**
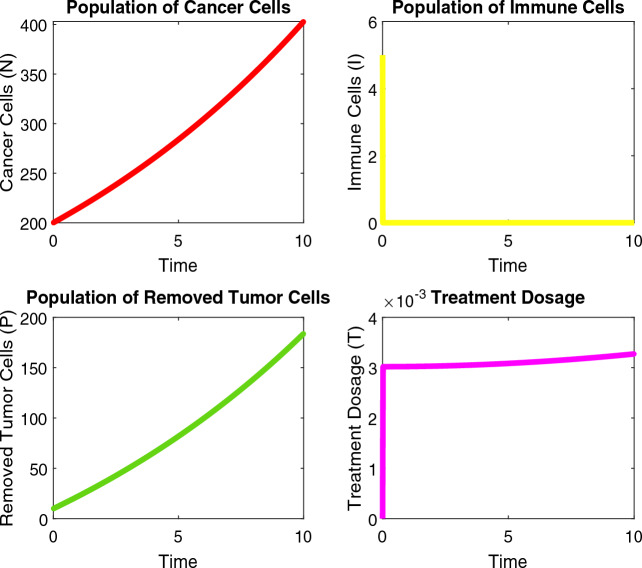
Treatment dosage via PID control.

**Figure 6:**
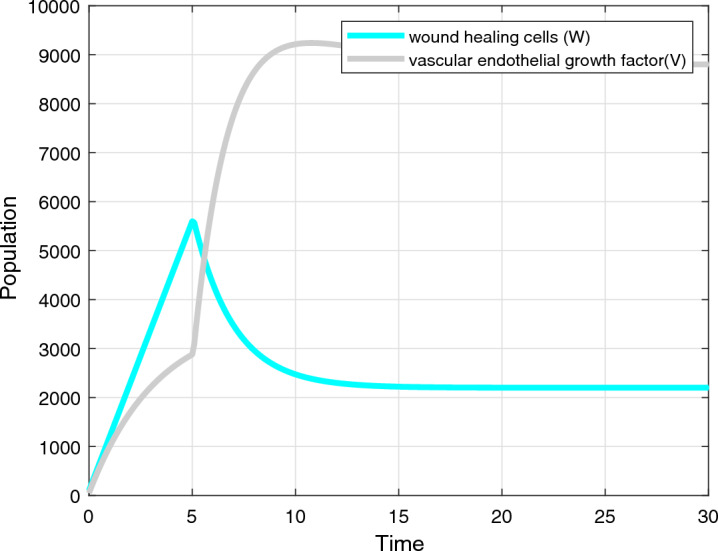
*W*(*t*) and *V*(*t*) plot.

**Figure 7:**
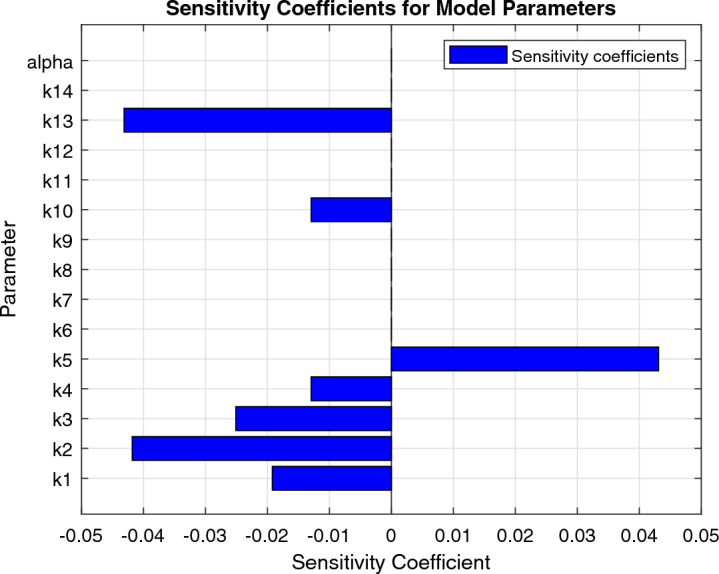
Sensitivity coefficients.

The numerical analysis is carried out using the Matlab FDE12 solver which implements the predictor–corrector method of Adams–Bashforth–Moulton. Values of parameters used through Figs. 2, 3, 4, 5, 6 and 7 include: $\lambda = 0.3$, $K = 10000$, $\mu = 0.01$, $\beta _1 = 0.01$, $\beta _2 = 0.01$, $\beta _3 = 0.04$, $\phi _1 = 0.03$, $\phi _2 = 0.04$, $\phi _3 = 0.01$, $\gamma = 0.07$, $\delta = 0.001$, $\alpha _1 = 0.5$, $\alpha = 0.7$, $\alpha _2 = 0.9$, $t_0 = 0$, $t_{\text {final}} = 400$, $h = 2^{-6}$, $N_0 = 3000$, $I_0 = 90$, $P_0 = 20$, $T_0 = 0$, $\phi _1 = 0.9$, $\phi _2 = 0.458$, $\phi _3 = 0.8$, $\phi _4 = 0.45$, $\phi _5 = 0.5$, $\phi _6 = 0.6$, $\phi _7 = 0.5$, $\phi _8 = 0.6$, $\phi _9 = 0.9$, $\phi _{10} = 0.5$, $\theta = 0.5$, $\eta = 0.7$, $\alpha _1 = 0.3$, $\tau = 5$, $t = 0:0.1:30$, $W_0 = 100$, $V_0 = 50$.

## Results and discussion

Analytical results show that the system has a unique positive equilibrium point, and is globally stable. The dynamics of the lung cancer model without treatment can be seen in Fig. 2. The simulation provides insights into the population dynamics of cancer cells (N), cancer cells that have spread (P), and immune cells (I) in the context of lung cancer. The different fractional order values ($\alpha$) of 0.5, 0.7, and 0.9 demonstrate varying effects on these populations. A notable observation is that higher fractional order values lead to faster convergence of the model, indicating improved accuracy and efficiency in describing the cancer dynamics. As ($\alpha$) increases, the growth rate of normal cells (N) decreases, suggesting a stronger inhibitory effect on cell proliferation. This implies that higher fractional order values enable the model to capture the control mechanisms governing cancer cell growth within lung tissue more effectively. Furthermore, the plot reveals that increasing ($\alpha$) results in a increase in the population of cancer cells that have spread (P), indicating an increased ability of cancer cells to form secondary tumors in other parts of the body. The faster convergence associated with higher fractional order values contributes to a more accurate representation of the suppression of metastasis, emphasizing the importance of incorporating a strong fractional derivative component in modeling the immune response against spreading cancer cells. In contrast, the immune cell population (I) exhibits fluctuating patterns, with oscillations becoming more pronounced as ($\alpha$) increases. This indicates that higher fractional order values lead to a more dynamic immune response, resulting in periodic fluctuations in the number of immune cells. The faster convergence of the model with higher fractional order values allows for a more precise depiction of the intricate interplay between immune cells and cancer cells, highlighting the complex dynamics of the immune system’s interaction with lung cancer.

The sensitivity coefficients for each parameter were calculated using the concept of the normalized sensitivity coefficient. The analysis indicated that the most sensitive parameter is the carrying capacity (*K*), followed by the growth rate of cancer cells ($\lambda$) and the rate at which cancer cells spread from the lung tissue to other parts of the body ($\mu$), as shown in Table 2 and Fig. 7. This suggests that interventions aimed at reducing the carrying capacity or inhibiting the growth or spread of cancer cells could be effective in reducing the number of cancer cells in the lung tissue and preventing their spread to other parts of the body. The analysis revealed that the parameter with the greatest impact on the steady-state solution of the system is the rate of tumor cell growth, $k_1$. Increasing $k_1$ by 1 percent would result in a 78.5 percent increase in the steady-state tumor cell concentration while decreasing the effectiveness of treatment by reducing $k_2$ by 1 percent would lead to a 130.7 percent increase in tumor cell concentration. The analysis also showed that the steady-state solution is most sensitive to changes in the rate of division of cancer stem cells ($k_5$) and the rate of differentiation of cancer stem cells into progenitor cells ($k_{13}$). Interventions that target cancer stem cells, such as therapies that inhibit stem cell division or promote their differentiation, may have a significant impact on the overall tumor cell population. The effects of immune cells, blood vessels, and growth factors were found to be less sensitive, indicating that these factors may play a less significant role in the growth and spread of lung cancer. However, this does not mean that they are not important, as they may still have important roles in specific stages or types of lung cancer. Overall, the sensitivity analysis provides valuable insights into the key parameters that drive the growth and spread of lung cancer and can inform the development of targeted interventions.

Furthermore, results show that the reproduction number $R_0$ represents the average number of cancer cells that are produced by a single cancer cell over the course of its lifetime. A value of 2.6 for lung cancer would indicate that, on average, each cancer cell would produce 2.6 (at least 2) new cancer cells during its lifetime as shown in Fig. 3. This can contribute to the rapid growth and spread of cancer within the body. The reproduction coefficient $R_C$ represents the rate at which cancer cells reproduce or divide, relative to the rate at which normal cells in the body reproduce or divide. A value of 0.22 for lung cancer would indicate that cancer cells are dividing at a rate that is 0.22 times the rate at which normal cells divide. This could suggest that cancer cells are growing more slowly than normal cells in the body, but it is important to note that cancer growth is a complex and multi-factorial process that involves many different factors beyond just cell division.

The result shows that the optimization models improve treatment, and dosage and reduce cancer growth. The objective function minimizes the population of cancer cells, tumor-promoting immune cells, and tumor cells that have not been surgically removed, and maximizes the population of surgically removed tumor cells, formulated using the weighting factors as seen in Fig. 4. The lung cancer optimization combined therapy model describes a modified system of equations that incorporates the effects of PDL1 monoclonal antibody immunotherapy and surgery as a control for lung cancer. The modified system of equations includes a term proportional to the product of treatment efficacy and the populations of cancer cells and immune cells to model the reduction of these populations due to the treatment. Additionally, the treatment has a time delay before it becomes effective.

The PID controller consists of three terms - proportional, integral, and derivative gains - each of which adjusts the dosage based on different factors. The proportional term adjusts the dosage based on the error between the target and the actual population of cancer cells, with larger errors resulting in larger adjustments. The integral term accumulates the error over time and adjusts the dosage to correct for consistent differences between the target and actual population. The derivative term adjusts the dosage based on the rate of change of the error, preventing overshooting of the target population. The magnitude of the adjustments for each term is proportional to the gain constant for that term - $K_p$ for the proportional term, $K_i$ for the integral term, and $K_d$ for the derivative term. By using the time evolution of the populations and the PID controller to adjust the dosage, the actual population of cancer cells can be brought within a specified tolerance of the target population, with the feedback control shown in Fig. 5. The target population of cancer cells can be set based on the stage and severity of the cancer, treatment goals, and desired outcomes, and may need to be adjusted based on patient response and changes in the cancer over time.

In Fig. 6, the *W*(*t*) plot demonstrates an initial surge in wound healing cells followed by a gradual decline, indicating the body’s acute response to tissue injury and subsequent resolution of the healing process. This suggests that the lung tissue’s healing capacity is dynamic, with an initial boost in wound-healing cells that gradually subsides as healing progresses. On the other hand, the *v*(*t*) plot reveals a sustained elevation of vascular endothelial growth factor, indicating a continuous need for angiogenesis to support tissue repair. This implies that the lung tissue requires a prolonged vascular supply to facilitate proper healing.

The employed approach has demonstrated high efficiency. The use of fractional order derivatives in this lung cancer model provides relevance by incorporating memory and non-local dependencies into the system. The fractional order ($\alpha$) influences the rate of change and interactions between different cell populations, representing the memory effects in cellular processes. By including fractional derivatives, the model captures the long-term behavior and complex interactions involved in lung cancer dynamics, contributing to a more accurate representation of the disease’s progression and potential therapeutic interventions. The novelty of this research lies in the development of a comprehensive fractional-order mathematical model for lung cancer that incorporates integrated therapeutic approaches. This approach allows for a more precise understanding of the intricate interactions among cancer cells, immune cells, and other constituents of the tumor microenvironment. Additionally, by incorporating PD-L1 monoclonal antibody treatment and surgery as controls in the model, this study can explore the potential benefits of combining these treatments and investigate their impact on tumor dynamics. This approach stands out from others because it offers a more comprehensive and accurate representation of lung cancer dynamics and treatment response. It also holds promise for guiding the development of novel treatment strategies for lung cancer and enhancing patient outcomes.

## Data Availability

All data generated or analyzed during this study are included in this published article.
